# Testing the effects of nicotine concentration and nicotine source claims on oral nicotine pouch packaging engagement, self-reported perceptions and use intentions, and product trial in young adults and adults: Protocol for a randomized trial

**DOI:** 10.1371/journal.pone.0362172

**Published:** 2026-11-06

**Authors:** Lauren Long, Mahmood A. Alalwan, Elsa Alvarado, Anna Kozar, Elise M. Stevens, Ce Shang, Megan E. Roberts, Elizabeth G. Klein, Abigail Shoben, Darren Mays

**Affiliations:** 1 Center for Tobacco Research, the Ohio State University Comprehensive Cancer Center, Columbus, Ohio, United States of America; 2 Division of Preventive and Behavioral Medicine, Department of Population and Quantitative Health Sciences, UMass Chan Medical School, Worcester, Massachusetts, United States of America; 3 Department of Internal Medicine, College of Medicine, The Ohio State University, Columbus, Ohio, United States of America; 4 Division of Health Behavior and Health Promotion, College of Public Health, The Ohio State University, Columbus, Ohio, United States of America; 5 Division of Biostatistics, College of Public Health, The Ohio State University, Columbus, Ohio, United States of America; PLOS: Public Library of Science, UNITED STATES OF AMERICA

## Abstract

**Background:**

Oral nicotine pouches (ONPs) are novel nicotine products and evidence on how features of ONP packaging affect product engagement, perceptions, use intentions, and behavior is limited.

**Objectives:**

We outline the protocol for a randomized trial examining how nicotine concentration and nicotine source claims affect engagement with ONP packaging using psychophysiological measures, self-reported ONP perceptions and use intentions, and ONP trial (ClinicalTrials.gov: NCT06192316).

**Methods:**

Between 2024 and 2027, we will recruit 450 participants, comprised of: 150 young adults ages 18–24 who are susceptible but do not use tobacco; 150 young adults ages 18–24 who use tobacco; and 150 adults ages 25–65 who use tobacco. Participants complete procedures at an in-person laboratory visit. During the visit, participants complete pre-exposure self-report measures and are randomized between subjects to one of nine conditions in a 3 (Nicotine Concentration: None, Low, or High) x 3 (Nicotine Source Claim: None, Tobacco Free, or Synthetic) design. Within conditions, participants view 16 ONP pack images, in random order, reflecting different ONP brands and flavors. During exposure, we collect psychophysiological measures including heart rate, skin conductance, and visual attention. After exposure, participants complete self-report measures of ONP perceptions and use intentions, and a timed behavioral task where they are presented with ONP packages corresponding to their randomized condition and brands and flavors shown in the stimuli. Participants can try one of the products, and ONP trial is the behavioral outcome of interest. However, the packages do not contain ONPs; they contain commercially available pouches with food grade ingredients. At visit conclusion, participants are de-briefed and receive their incentive for participating.

**Conclusions:**

This project will generate evidence on the effects of nicotine concentration and source claims on ONP packaging in young adults who are not using but susceptible to tobacco, young adults using tobacco, and adults using tobacco to inform potential regulations*.*

## Introduction

Oral nicotine pouches (ONPs) are small, pre-portioned pouches placed between the lip and gum that deliver nicotine without tobacco leaf or combustion, and they are available in multiple nicotine concentrations and flavors [1]. Since entering the U.S. market in 2016, ONP sales have increased rapidly [2–4]. Tobacco and nicotine products exist along a continuum of harm, with combustible products such as cigarettes generally causing substantially greater health risks than non-combustible nicotine products [5]. This risk continuum is important for evaluating the potential public health impact of ONPs [6]. Current evidence indicates that ONPs contain substantially lower levels of toxicants than many tobacco products, including cigarettes, and may have a toxicant profile similar to nicotine replacement therapy [6–9]. Thus, ONPs could reduce health risks for adults who smoke cigarettes if they switch completely from cigarettes to ONPs or use ONPs as a means of quitting smoking.

However, the population level impact of ONPs will depend on their appeal, uptake, and patterns of use across different groups. ONPs may provide potential benefit for adults who smoke, but ONP use by young people who do not currently use tobacco and nicotine could increase nicotine exposure and risk for nicotine dependence. This creates a regulatory challenge: product features that may encourage switching among adults who use combustible tobacco may also increase appeal, reduce perceived risk, or encourage trial among young people.

Packaging is a primary vehicle for tobacco and nicotine product marketing and can influence product appeal, risk perceptions, and intentions to use tobacco and nicotine products [7–9]. ONP packaging commonly emphasizes nicotine concentration (e.g., 2 mg, 6 mg) and includes nicotine source claims such as “tobacco free” or “synthetic” nicotine [10–13]. These nicotine-related packaging features may shape how consumers understand the product [14]. Nicotine concentration labels may signal product strength, satisfaction, or addiction potential, whereas nicotine source claims may influence perceptions of risk, addictiveness, and the product’s relationship to conventional tobacco products. However, the public health impact of these packaging features may differ across populations. Adults who currently use tobacco and nicotine may interpret these features as indicators of product substitution or harm reduction, whereas non-using young adults who are susceptible to tobacco and nicotine use may interpret the same features as indicators of safety, novelty, or acceptability.

Despite the increasing availability and marketing of ONPs, there is little experimental evidence on how nicotine-related packaging features affect consumer responses to these products and limited evidence to date is mixed. For example, Ma and colleagues [15] tested the effects of modified risk claims, nicotine concentration, and nicotine source claims in information about ONPs in adults who smoke cigarettes. They found that lower nicotine concentration and tobacco free claims were perceived to be less harmful. In contrast, Mays and colleagues [16] found in an experimental study with adults who use tobacco and those who do not that ONP packages with higher nicotine concentration were viewed as less harmful and less addictive. To our knowledge, no study has comprehensively tested how nicotine concentration and nicotine source claims on ONP packaging influence psychophysiological, self-report, and behavioral responses in young adults who are susceptible but do not use tobacco and nicotine, young adults who use tobacco and nicotine, and adults who use tobacco and nicotine. This evidence can inform regulatory measures surrounding ONP packaging by better understanding the potential public health effects of ONP packaging features.

We report the protocol for a single-visit, between-subjects randomized trial designed to test the effects of nicotine concentration and nicotine source claims on responses to ONP packaging. Participants will be randomized to view ONP pack images that vary by nicotine concentration and source claim. During exposure, we will assess psychophysiological engagement with ONP packaging. After exposure, we will assess ONP risk perceptions, intentions to use ONPs, and ONP trial using a behavioral task. The study will enroll young adults who are susceptible but do not use tobacco and nicotine, young adults who use tobacco and nicotine, and adults who use tobacco and nicotine. Findings will advance tobacco regulatory science by identifying whether nicotine related ONP packaging features influence psychophysiological, self-report, and behavioral outcomes among populations with different levels of vulnerability and potential benefit.

## Materials and methods

### Design overview and setting

This is a single visit, 3 x 3 between subjects randomized trial at the Center for Tobacco Research at The Ohio State University Comprehensive Cancer Center in Columbus, Ohio. The trial is designed to test the effects of nicotine concentration (none on label, low, high) and nicotine source claims (None, “Tobacco Free,” “Synthetic Nicotine”) on psychophysiological, self-report, and behavioral outcomes. We will enroll 450 participants across three tobacco and nicotine use groups (young adults who are susceptible but do not use tobacco and nicotine, young adults who use tobacco and nicotine, and adults who use tobacco and nicotine). Participants will view 16 ONP package images while eye-tracking, electrocardiographic, and electrodermal activity data are collected. They will then complete self-report measures and a behavioral task assessing ONP trial. An overview of the study design, measures, and outcomes is shown in Fig 1.

**Fig 1:**
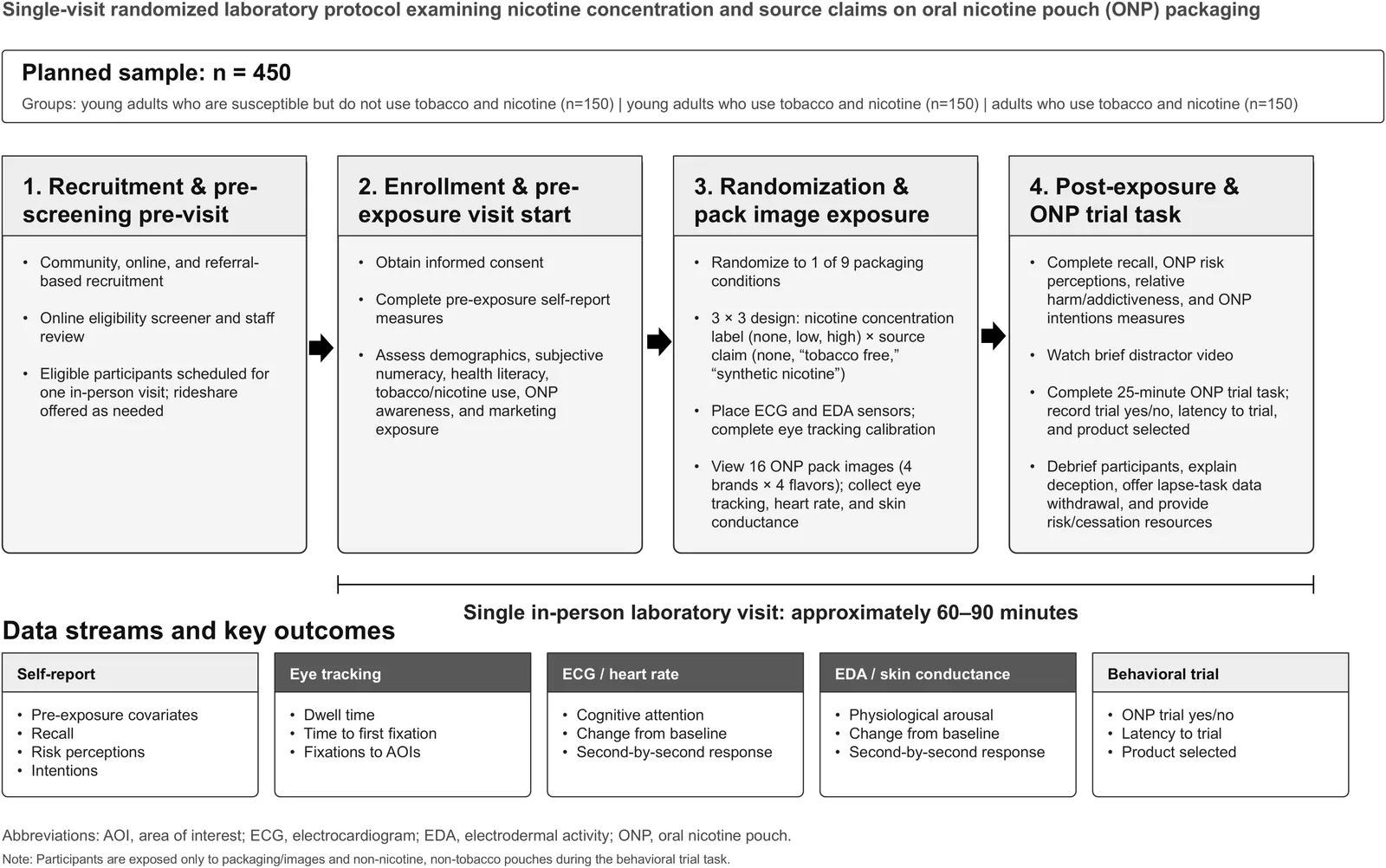
Participant timeline and schedule of study procedures.

### Objectives and hypotheses

The trial will address two primary objectives: 1) To examine the effects of nicotine concentration and source claims on engagement with ONP packaging using psychophysiological measures captured during exposure to ONP pack images and 2) To examine the effects of nicotine concentration and source claims on self-report ONP perceptions and behavioral intentions, and ONP trial. We hypothesize that participants randomized to higher nicotine concentration and “tobacco free” claims will have increased visual and cognitive engagement with ONP packaging. We further hypothesize that the higher nicotine concentration and “tobacco free” claims will decrease perceived risks of ONPs, increase intentions to use ONPs, and increase ONP trial. We hypothesize that these effects will differ by tobacco and nicotine use group, with stronger effects among adults and young adults who use tobacco and nicotine than young adults who are susceptible but do not use tobacco and nicotine.

### Participants

#### Planned enrollment and participant groups.

Participant recruitment started on March 04, 2024, and is ongoing. We plan to enroll 450 participants: 150 young adults ages 18–24 who are susceptible but do not use tobacco and nicotine, 150 young adults ages 18–24 who currently use tobacco and nicotine, and 150 adults ages 25–65 who currently use tobacco and nicotine. We anticipate completing participant recruitment and data collection by August 31, 2027. Data analysis will follow completion of data collection, and study results are expected to be available in January 2028. During recruitment, we will track tobacco and nicotine susceptibility and use subgroups to position the trial for exploratory analyses based on subgroups of participants who are susceptible to or currently use specific tobacco and nicotine products. For young adults who are susceptible but do not use tobacco and nicotine, subgroup classification will be based on the product categories to which participants are susceptible: combustible products (e.g., cigarettes, cigars) only, noncombustible products (e.g., smokeless tobacco, e-cigarettes) only, or products in more than one category. For young adults and adults who currently use tobacco and nicotine, subgroup classification will be based on current use of combustible products only, noncombustible products only, or products in more than one category.

#### Eligibility criteria.

Eligible participants must meet criteria for one of the three participant groups, be willing and able to complete an in-person laboratory visit lasting approximately 60–90 minutes, and they must be able to read and speak English. Young adults susceptible to tobacco and nicotine use must report no current tobacco and nicotine use and meet the susceptibility criteria related to using cigarettes, cigars, waterpipe/hookah, smokeless tobacco, e-cigarettes, ONPs, and any other tobacco and nicotine products specified by participants. For each product, susceptibility is assessed with a valid, 3-item measure capturing if they are curious about the product, would try the product soon, or would try the product if given one by a friend [17–21]. Response options include definitely yes, probably yes, probably not, and definitely not. Participants are classified as susceptible to a product if they provide any response other than “definitely not” to at least one of the three items.

For current tobacco and nicotine use, we assess lifetime use of cigarettes, cigars, waterpipe/hookah, smokeless tobacco, e-cigarettes, ONPs, and other participant-specified tobacco and nicotine products. Participants who report lifetime use are asked whether they currently use each product every day, some days, or not at all. If they indicate using a product every day or some days, we assess duration of use. For eligibility, we define current tobacco and nicotine use as using any of the tobacco and nicotine products every day or some days for 6 months or longer.

To minimize prior familiarity with ONPs, participants must either have never used ONPs or have used ONPs no more than 10 times in their lifetime, with no ONP use during the previous three months. Exclusion criteria are age younger than 18 years or older than 65 years; failure to meet criteria for one of the three participant groups; inability or unwillingness to complete the laboratory visit; ONP use during the previous three months; or ONP use more than 10 times in the lifetime.

#### Recruitment and screening.

We are recruiting participants through social media advertising, community-based advertising, word of mouth referrals, re-contact of participants from prior related studies who agreed to future contact, and direct outreach to potentially eligible university students. Recruitment materials direct interested participants to a study website and online eligibility screener. Potential participants may also contact the study team to ask questions or complete screening with staff assistance.

Study staff review responses to the online screener to verify eligibility. This is followed by contacting respondents to administer the screener verbally to clarify any questions about online screener responses and confirm eligibility. Once eligibility is confirmed, research staff schedule participants for an in-person laboratory visit. Rideshare transportation to and from the study visit is provided to eligible participants who live within reasonable driving distance but otherwise lack transportation.

### Study materials

#### Digital ONP package stimuli.

We developed ONP pack images for the study by analyzing data on ONP sales in the U.S. based on product features, including brands, flavors, and nicotine concentration (high, low) for each brand [4]. We also examined the existing literature [10–13] and analyzed data from online retailers on prominent marketing claims [22]. This increases external validity of the stimuli for study conditions relative to ONPs available in the U.S. market.

Using these data, we selected the top four most common ONP brands (Zyn, On!, Rogue, Velo) and flavors (mint, wintergreen, citrus, cinnamon). We chose to vary brand and flavor in order to ensure that observed differences by nicotine claims were not attributable to brand- or flavor-specific effects. We obtained front-facing images of packages for each ONP product (i.e., by brand, flavor, and nicotine concentration) from online retailers. We digitally edited pack images to create nine experimental versions of each image corresponding to conditions in the 3 × 3 factorial design. We selected low and high concentration labels to represent the lowest and highest concentrations available in the market for each brand. We also standardized nonexperimental package features, including image dimensions, label placement, color presentation, and removed extraneous promotional information such as loyalty-rewards messaging. The result of this process was a total of 144 digital ONP pack images, corresponding to the 3 by 3 experimental design, with images for 4 ONP flavors for each brand, 4 brands for each condition, and 9 conditions total. The final pack image stimuli are available from the corresponding author.

#### Physical packaging and pouches.

For the behavioral task capturing ONP trial, we used the digital images developed above to create physical ONP packaging corresponding to each experimental condition in the design. To do so, we purchased ONPs for each brand and flavor, removed the pouches and labels, sanitized containers, and applied condition-specific adhesive labels. Before printing, label colors were adjusted to match the colors of the original product packaging. We filled the relabeled containers with commercially available tobacco- and nicotine-free pouches containing food grade flavorings and fillers but no nicotine, tobacco, caffeine, or other active ingredients. This design was chosen so that we could assess a real behavioral outcome (ONP trial), while not exposing participants to the risks of an addictive product. We conducted chemical testing to confirm that the pouches contained no detectable nicotine or nicotine analogs.

### Procedures

#### Overview.

Each participant will complete one in-person laboratory visit lasting approximately 60–90 minutes. At the start of the visit, study staff will obtain informed consent. Eligible, consenting participants will complete self-report pre-exposure measures, undergo sensor placement and eye-tracker calibration, view ONP pack images corresponding to their assigned condition while psychophysiological outcomes are captured, complete post-exposure self-report outcome measures including a recall task, and complete the behavioral task assessing ONP trial. Staff then will debrief participants and provide compensation.

Self-report measures will be administered using Research Electronic Data Capture (REDCap). Self-report measures use valid assessments and align with the PhenX Toolkit [23–25] where possible to maximize reproducibility. Psychophysiological measures including eye tracking, electrocardiographic (ECG), and electrodermal activity (EDA) data will be collected using iMotions software and associated equipment.

#### Pre-exposure measures.

Before package exposure, participants will complete measures of sociodemographic characteristics, including sex, gender, age, race and ethnicity, education, employment status, household income, subjective financial situation, and family socioeconomic status [23–27]. Participants will also complete measures of subjective numeracy [28], BRIEF health literacy [29], tobacco- and nicotine-related beliefs, lifetime and current tobacco and nicotine use, product-specific frequency and duration of use, usual brands, nicotine dependence, ONP awareness, and previous exposure to ONP marketing [23–25].

#### Randomization and masking.

After completing pre-exposure measures, participants will be randomly assigned in equal proportions to one of the nine between subjects experimental conditions in the 3 (Nicotine Concentration: None on label, Low, High) x 3 (Nicotine Source Claim: None, “Tobacco Free,” “Synthetic Nicotine”) factorial design. The study biostatistician (AS) will generate the random allocation sequence before enrollment using permuted blocks stratified by tobacco and nicotine susceptibility and use group (young adults who are susceptible but do not use tobacco and nicotine, young adults who use tobacco and nicotine, and adults who use tobacco and nicotine) for balance across the nine conditions. Assignment will be implemented through REDCap.

Participants cannot be masked to the packages they view, but they will not be informed of the specific hypotheses. Staff responsible for implementing visits cannot be practically blinded to participants’ assigned conditions.

#### Package exposure and psychophysiological measures.

Participants are seated in front of a high-resolution monitor at a comfortable viewing distance (60–70 cm). Visual attention is recorded using SmartEye Aurora remote eye tracker (Smart Eye AB, Gothenburg, Sweden) mounted on the monitor lower bezel. Study staff place four pre-gelled ECG electrodes and two EDA sensors on the forearms and fingers, respectively. Participants are instructed to remain as still as possible during recording.

Before beginning the study, participants complete a practice run using a single image to familiarize them with the study flow and eye-tracker calibration process. After the practice run, the eye tracker is recalibrated as part of the actual study procedures before participants view the experimental ONP pack images. Calibration may be repeated up to three times if an acceptable calibration is not achieved. These two calibration procedures, one during the practice run and one before the experimental image viewing task, are conducted to ensure eye-tracking data quality.

Participants view 16 ONP pack images representing each brand–flavor combination and 16 beverage images that serve as a washout between ONP images. All ONP images display the nicotine concentration and source claim corresponding to the participant’s assigned experimental condition. Stimulus presentation alternates between ONP and beverage images and within each product category, the order of the 16 images is randomized.

Each image is preceded by a 5-second black screen displaying the words “Get Ready” to return psychophysiological measures to baseline and is then presented for 10 seconds. In total, the task includes 32 image presentations and 32 baseline intervals. The “Get Ready” intervals are analyzed as physiological baselines to assess change in ECG and EDA during the subsequent image presentation. Eye-tracking, ECG, and EDA data are recorded continuously in iMotions (Version 11). The complete image-viewing task lasts 8 minutes.

#### Post-exposure measures.

Immediately after package exposure, participants complete open-ended recall questions and self-report measures assessing ONP risk perceptions, comparative harm and addictiveness, curiosity, interest, and likelihood of future use. Detailed outcome definitions are provided below.

#### ONP trial task.

After completing post-exposure self-report measures, participants watch a brief distractor video (e.g., six-minute video about nature) and then complete a behavioral task assessing ONP trial. The trial task is a valid behavioral measure that was originally developed for smoking cue reactivity research and used in prior work by others [30–33]. The task lasts 25 minutes in duration and is designed to disincentivize immediate trial to provide participants with the opportunity to interact with the products and their packaging, as described below.

In the visit room, participants will be presented with ONP packages with labels modified to correspond to the nicotine concentration and nicotine source claim condition to which they are randomized. Packages also correspond to the brands and flavors shown in the stimuli they viewed previously. Participants are instructed that they can try one of the products at any time, but they can earn $0.20 for every 1 minute they delay trying, totaling $5 if they abstain for the full 25-minute period. Participants are instructed that the potential to continue to earn money will be eliminated at the time they attempt to use any of the ONPs [30,31], and that they are required to remain in the room for the full 25 minutes regardless of trial of the ONPs.

Participants are instructed not to use their phones or other personal devices during this time. Staff monitor participants from a separate control room. When participants attempt to use any of the ONPs by placing the pouch in their mouth, study staff stop the task, record the outcome as a trial, record the time of the trial, and which product they tried. For participants who do not try an ONP for the entire 25 minutes, study staff record the outcome as a non-trial. Staff also record notes about participant interactions with the packaging throughout the task.

#### Debriefing and participant compensation.

Immediately after the behavioral task, staff disclose that the containers held tobacco- and nicotine-free pouches and that all participants will receive the maximum $5 incentive, added to their total incentive, regardless of their behavior. Staff explain the scientific rationale for the IRB-approved incomplete disclosure and provide participants an opportunity to decline retention of their behavioral-task data. All participants receive information about the health risks of tobacco and nicotine products and available cessation resources. After completing all study procedures, participants receive a total of $105.

### Measures and outcomes

#### Visual attention.

Visual attention is measured via eye movements using SmartEye Aurora (https://smarteye.se/aurora/) eye tracker with a 60 Hz sampling rate. Areas of interest (AOIs) are predefined for each pack image including: 1) Full pack image; 2) Nicotine concentration; 3) Nicotine source claim; 4) Branding (i.e., brand name, flavors); 5) Warning label. For images without a concentration label or source claim, spatially matched regions are defined so that gaze behavior can be compared across conditions. AOIs will not overlap.

The primary outcomes for visual attention are AOI dwell time, time to first fixation, and fixation (i.e., saccade) count [34–36]. Dwell time is the total duration of valid gaze within an AOI; time to first fixation is the elapsed time from image onset to the first fixation within an AOI; and fixation count is the number of discrete fixations within an AOI. Proportion of viewing time directed to each AOI will also be calculated. Images for which a participant never fixates on an AOI will be treated as censored at the end of the image presentation for time to first fixation analyses.

#### Cognitive resources allocated to message processing.

Conceptualized as the cognitive resources allocated to encode a message into working memory, this measure will be operationalized as the difference in heart rate (HR) between a baseline period prior to each pack image and during each second of the pack image exposure [37–40] assessed with ECG. Beats per minute (BPM) are collected with the Shimmer3 sampled at 512 Hz. Greater heart-rate deceleration relative to baseline will be interpreted as greater allocation of cognitive resources to stimulus processing. This is an established psychophysiological measure of cognitive resources devoted to stimuli processing [37–40].

#### Physiological arousal.

Arousal is an indicator of physiological activation of the sympathetic nervous system but does not by itself indicate whether a response is positive or negative. It is measured using EDA recorded with Shimmer3 Galvanic Skin Response sensors sampled at 128 Hz [40]. Similar to heart rate, arousal will be operationalized as the difference in EDA between a baseline period prior to each pack image and during each second of the pack image exposure [37–40].

#### Recall.

We capture unaided recall, a measure of storage in memory [41], of nicotine concentration and source information corresponding to conditions to which participants are randomized with open ended questions. Responses will be coded, and coding reliability will be established, to create a measure of recall of information about nicotine dimensions in the stimuli [42,43].

#### ONP perceptions.

We capture perceptions of ONPs with 7 items that align with recommendations for assessing cognitive and affective aspects of tobacco and nicotine risk perceptions [44]. Items assess perceived risk of health harm from ONPs, perceived risk of addiction from ONPs, worry about harm, and worry about addiction [45–48]. The items are on a 1–7 response scale, have excellent reliability, and are averaged to create an overall score. We also capture perceived harm and addictiveness of ONPs relative to cigarettes, smokeless tobacco, and e-cigarettes using valid items [43,49,50].

#### ONP intentions.

We will assess curiosity, interest, and likelihood of using ONPs post-exposure. These are valid predictors of future use of novel tobacco and nicotine products among never users [46,48,51]. Items are on a 1–7 scale, have excellent reliability, and are averaged to create an overall ONP intentions score, with higher scores indicating stronger intentions to use ONPs.

#### ONP trial.

The primary behavioral outcome is whether the participant places a pouch in the mouth during the behavioral task (yes/no).

### Statistical analysis

Analyses will follow participants’ randomized assignments. We will summarize participant characteristics overall and by experimental condition and participant group. Baseline characteristics will be examined descriptively to identify substantial imbalances for covariate adjustment. All tests will be two-sided with α = .05, and effect estimates will be reported with 95% confidence intervals. Analyses will be conducted using SAS (Version 9.4).

All primary models will include fixed effects for nicotine concentration, nicotine source claim, participant group, the concentration × source claim interaction, and the interaction of each experimental factor with participant group. Participant group will be modeled as a three-level factor comprising young adults who currently use tobacco and nicotine, adults who currently use tobacco and nicotine, and young adults who are susceptible but do not use tobacco and nicotine. The concentration × source claim × participant group interaction will be evaluated as a secondary prespecified analysis.

For continuous image-level outcomes, including dwell time, proportion of valid viewing time, heart-rate change, and EDA change, we will fit linear mixed-effects models with repeated observations nested within participants. Models will include brand, flavor, and presentation order as fixed effects and a participant-specific random intercept; additional random effects will be included when supported by model fit. Skewed outcomes will be transformed or analyzed using an appropriate generalized mixed model. Fixation count will be analyzed using a Poisson or negative binomial mixed model, depending on dispersion. Time to first fixation will be analyzed using a time-to-event model that treats images without a fixation in the AOI as censored at image offset.

Primary analyses of ECG and EDA outcomes will use prespecified image-level summary measures calculated relative to the baseline period preceding each image. Time-course analyses will model physiological responses across the 10-second image presentations nested within participants. Models of EDA will account for the expected latency between stimulus onset and the electrodermal response. Psychophysiological data will be pre-processed and cleaned according to published standards [35,36,40]. Prespecified signal-quality criteria will be applied at the signal-segment, image-presentation, and participant levels, as applicable.

Participant-level continuous outcomes, including ONP risk-perception and intention scores, will be analyzed using linear regression models. Binary recall outcomes and behavioral trial will be analyzed using logistic regression.

Model assumptions will be evaluated using appropriate diagnostic procedures. If assumptions are materially violated, models fail to converge, or the prespecified model is otherwise inappropriate for the observed data distribution, alternative transformations, distributional assumptions, link functions, covariance structures, or modeling approaches will be considered as appropriate. Any deviations from the prespecified analytic approach will be documented and justified, and sensitivity analyses will be conducted when feasible to assess the robustness of findings to alternative model specifications.

Missing self-report data will not be imputed. Psychophysiological observations that fail prespecified signal-quality thresholds will not be imputed; analyses will use the remaining valid observations. The amount and reasons for missing or excluded data will be summarized by experimental condition, and sensitivity analyses will assess the potential influence of differential data loss.

### Sample size

Our analyses will examine the effects of nicotine concentration and source claims on engagement with ONP packaging based on psychophysiological measures and their effects on post-exposure measures of ONP perceptions, behavioral intentions, and ONP trial. In both, we will examine if nicotine concentration and source claims differentially affect young adults who are susceptible but do not use tobacco and nicotine and adults who use tobacco and nicotine. Our target sample is 450 participants, including 150 young adults who are susceptible but do not use tobacco and nicotine, 150 young adults who use tobacco and nicotine, and 150 adults who use tobacco and nicotine. For a 3 (Nicotine Concentration: None on label, Low, High) x 3 (Nicotine Source Claim: None, “Tobacco Free,” “Synthetic Nicotine”) between-subjects design with 16 repeated measures, *α* = .05, and *r* among repeated measures of .5, this sample provides >80% power to detect small (*f* = .11) mean differences between levels for nicotine concentration or source claim by tobacco and nicotine use status. These effect sizes are consistent with our prior work [40] and psychophysiological research generally [52]. For other outcomes, our sample positions us to detect effects similar to our prior work examining how features of tobacco and nicotine marketing (e.g., advertising, packaging) affect risk perceptions, behavioral intentions, and other outcomes.

### Data management

All study materials will be kept on The Ohio State University Comprehensive Cancer Center secure computer network behind a firewall and password protected. Study tracking and self-report data will be collected, managed, and secured using the REDCap platform, which will be accessible only to authorized study personnel. Psychophysiological measures are collected using iMotions with participants’ study identifiers only (i.e., no personally identifying information) and securely stored on the university network. All computer systems will be password-protected to prevent unauthorized access, and all network-based inter-site communications of confidential data will be encrypted. Participants will be assigned a unique identifier which will be used to identify them in all study data. Participant information will be accessible only to authorized research staff who are pledged to confidentiality and have received training in the ethical conduct of research.

### Safety considerations

The primary potential source of risks in this study is the social risk of gathering behavioral information about tobacco and nicotine use and related attitudes, perceptions, and beliefs. These potential risks include fear of disclosure of potentially sensitive information about tobacco and nicotine use. Some people may feel uncomfortable answering questions about their tobacco and nicotine use history or use status. Informed consent will be obtained from all participants prior to study enrollment, emphasizing the voluntary nature of the study. Participants may skip any questions that may make them uncomfortable aside from demographic questions pertaining to eligibility.

### Data sharing and dissemination

All dataset(s) that can be shared will be deposited in the Inter-university Consortium for Political and Social Research (ICPSR). ICPSR will make the research data from this project available to the broader social science research community as public-use data files. These files, in which potential direct and indirect identifiers have been removed to minimize disclosure risk, may be accessed directly through the ICPSR website. Data files will be findable and identifiable by assignment of a persistent unique identifier (a DOI) upon their availability in the repository. After agreeing to Terms of Use, users with an ICPSR MyData account and an authorized IP address from a member institution may download the data, and non-members may purchase the files. Data from this project will be supplied to ICPSR at the time of publication of the relevant results or at the end of the project period, whichever is sooner.

### Ethical declarations

This study has been registered with clinicaltrials.gov under the identifier NCT06192316, submitted 12/13/2023. This study was initially reviewed and approved by the Ohio State University Institutional Review Board under Study Number 2023C0092 on 07/05/2023. The current version of the protocol (version 4) was reviewed and approved on 10/02/2025 and is available as Supporting Information. Informed consent, including a legally valid signature, is obtained using both verbal and written descriptions of the study and REDCap system. Participants are clearly informed that their participation is voluntary and that they may withdraw at any time. An electronic signed copy of the consent form is provided to participants at the time of consent for their records. Only IRB-approved study personnel are responsible for obtaining consent. Any modifications to the study protocol are submitted to the OSU Cancer IRB and ClinicalTrials.gov, and such changes will be documented in future publications as necessary.

## Discussion

As sales of ONPs continue to climb, [4] there is a pressing need for data to inform policy and regulation. This includes a need for evidence on how prominent features of ONP marketing affect consumers, including those who do not use and those who use tobacco and nicotine. This trial is designed to comprehensively examine the effects of nicotine concentration and nicotine source claims on ONP packaging with a rigorous, comprehensive set of measures including self-report, psychophysiological, and behavioral outcomes. We will enroll young adults who are susceptible but do not use tobacco and nicotine, young adults who use tobacco and nicotine, and adults who use tobacco and nicotine to assess the potential public health effects of ONP packaging features.

Our trial has important limitations to acknowledge. First, the trial is conducted with a convenience sample recruited for an in-person study in a single geographic location. This may reduce generalizability of our findings to broader populations. Second, we designed our trial and our stimuli based on data inputs on the most prominent ONP brands and flavors in the U.S. However, as the number of ONP brands available in the U.S. continues to grow, future work to replicate and extend this study to other ONP brands will be important. Third, although we are studying two prominent features of ONP packaging (nicotine concentration and nicotine source claims), emergent features introduced as manufacturers diversify packaging (e.g., using qualitative indicators of nicotine concentration rather than quantitative information) should also be examined in future studies. Finally, all procedures occur at a single study visit, with no prospective follow-up. Future studies can build from this work by assessing behavioral impacts of ONP packaging features prospectively.

Our trial also has notable strengths. We are using a rigorous experimental design, and our approach to stimuli development leverages data on the most popular ONPs to balance experimental control with external validity. Importantly, our experimental design accounts for product features other than nicotine concentration and nicotine source claims that may influence responses to ONP packaging (e.g., brand, flavor). We are also capturing a comprehensive set of outcome measures, including psychophysiological responses during exposure to the ONP pack images, self-report outcomes, and behavioral responses. Our recruitment and sampling approach is also a strength. We will enroll young adults who are susceptible but do not use tobacco and nicotine, young adults who use tobacco and nicotine, and adults who use tobacco and nicotine to capture the potential public health effects of ONP packaging features.

Results of this trial are poised to advance tobacco regulatory science and inform tobacco regulation regardless of whether our hypotheses are supported. Findings of this study can inform regulators and policymakers regarding ONP packaging features that can be addressed to maximize the potential public health benefits of ONPs (e.g., to adults who smoke) and minimize the potential risks to public health (e.g., to young adults who do not use tobacco and nicotine). Such evidence surrounding the public health effects of ONP marketing features is sorely needed as ONP sales continue to climb, and regulators and policymakers need data to inform decisions about how to address them from a public health perspective.

## Supporting information

S1 Protocol Human laboratory study of the effects of nicotine product claims on appeal, perceptions, and use behavior. (DOCX)

S1 Checklist SPIRIT 2025 editable checklist. SPIRIT 2025 checklist of items to address in a randomized trial protocol.
(DOCX)
