# Supplementary material for: Testing the effects of nicotine concentration and nicotine source claims on oral nicotine pouch packaging engagement, self-reported perceptions and use intentions, and product trial in young adults and adults: Protocol for a randomized trial
Source: PLoS One. 2026 Nov 6;21(11):e0362172. doi: 10.1371/journal.pone.0362172 (PMC13742948; doi:10.1371/journal.pone.0362172)
Supplement: Supplementary file 1 [file pone.0362172.s001.docx]

Human Laboratory Study of the Effects of Nicotine Product Claims on Appeal, Perceptions, and Use Behavior

***Principal Investigator(s):***

**Dr. Darren Mays, PI**

Division of Medical Oncology

Department of Internal Medicine

Cancer Control Program

OSUCCC Center for Tobacco Research

***Co-Investigator(s):***

**Dr. Megan Roberts,** OSU

**Dr. Ce Shang,** OSU

**Dr. Elizabeth Klein,** OSU

**Dr. Abigail Shoben,** OSU

OSU-23115

IRB Protocol Number: 2023C0092

Version: #4

IRB Approval Date: 10/2/2025

| **Revision #** | **Revision Summary** | **Protocol Version date** | **Consent Form Version Date** | **IRB Approval**  **Date** |
| --- | --- | --- | --- | --- |
| NA | Original submission | **05/30/2023** |  |  |
| 1 | Updated protocol to remove an unused measure and changed language for ECG sensors and lapse task | **11/27/2023** | **11/27/2023** | **12/21/2023** |
| 2 | Updated inclusion/exclusion criteria to allow those who have tried ONPs to be eligible for the study. Given the increased awareness and use of ONPs, we will begin allowing people who have tried ONPs greater than 3 months ago and have only used them 10 times or less to be eligible as long as they meet all other criteria. Corrected the name of the SmartEye eye tracker we are using: the Aurora eye tracker. Added BRIEF health literacy to the measures section 4.3. Added the Registrar office as a recruitment channel. | **09/04/2024** | **09/04/2024** | **10/22/2024** |
| 3 | Updated recruitment table and plans to align with original grant application. Updated measures to include items capturing beliefs about nicotine. | **08/26/2025** | **09/04/2024** | **10/2/2025** |

Table of Contents

[Abbreviations	3](#_Toc133565492)

[1.	Study Summary	4](#_Toc133565493)

[2.	Study Purpose & Aims	4](#_Toc133565494)

[2.1	Aims & Expected Findings	4](#_Toc133565495)

[3.	Background, Rationale, and Significance	5](#_Toc133565496)

[3.1	Background	5](#_Toc133565497)

[3.2	Significance	5](#_Toc133565498)

[3.3	Rationale	6](#_Toc133565499)

[3.4	Innovation	9](#_Toc133565500)

[4.	Study Design & Measures	10](#_Toc133565501)

[4.1	Research Design Overview	10](#_Toc133565502)

[4.2	Experimental Procedures	10](#_Toc133565503)

[Psychophysiological	10](#_Toc133565504)

[Lapse Task	11](#_Toc133565505)

[Debriefing	12](#_Toc133565506)

[4.3	Measures	12](#_Toc133565507)

[4.4	Subject Compensation	13](#_Toc133565508)

[5.	Subject Selection	14](#_Toc133565509)

[5.1	Inclusion & Exclusion	14](#_Toc133565510)

[5.2	Justification for Inclusion & Exclusion	14](#_Toc133565511)

[5.3	Recruitment	14](#_Toc133565512)

[5.4	Number of Participants	15](#_Toc133565513)

[5.5	Feasibility	15](#_Toc133565514)

[6.	Study Procedures	16](#_Toc133565515)

[6.1	Eligibility Screening	16](#_Toc133565516)

[6.2	Consent Process	16](#_Toc133565517)

[6.3	Subject Registration	16](#_Toc133565518)

[6.4	Experimental Study	16](#_Toc133565519)

[7.	Data Collection and Management	17](#_Toc133565520)

[7.1	Safeguards for subject confidentiality	17](#_Toc133565521)

[7.2	Record Retention	17](#_Toc133565522)

[8.	Risks and Benefits	17](#_Toc133565523)

[8.1	Risks and Benefits	17](#_Toc133565524)

[8.2	Minimizing Risks and Benefits	18](#_Toc133565525)

[8.3	Risk Justification	18](#_Toc133565526)

[9.	Adverse Event Reporting	18](#_Toc133565527)

[10.	Statistical Considerations and Data Analysis	19](#_Toc133565528)

[10.1	Power and Sample Size	19](#_Toc133565529)

[10.2	Statistical Analyses	19](#_Toc133565530)

[11.	References	20](#_Toc133565531)

# Abbreviations

**AE:	Adverse Events**: is an undesired harmful effect resulting from a medication or other intervention / study procedure.

**CDC**:	**Centers for Disease Control and Prevention**: the nation’s leading science-based, data-driven, service organization that protects the public’s health

**ECG**:	**Electrocardiogram**

**EDA**:	**Electrodermal activity**

**FDA:	Food and Drug Administration**: is a federal agency responsible for protecting and promoting public health through the regulation and supervision of tobacco products and other products.

**FSPTCA:	Family Smoking Prevention and Tobacco Control Act**: is a federal statute signed into law by President Barack Obama on June 22, 2009 that gives the FDA the power to regulate the tobacco industry.

**GSR**:	**Galvanic Skin Response**

**HRV:	Heart Rate Variability**

**IRB**:	**Institutional Review Board**

**NCI:	National Cancer Institute**: part of the U.S. National Institutes of Health.

**NIH:	National Institute of Health**: an agency of the United States Department of Health and Human Services.

**ONP**:	**Oral Nicotine Pouch:** white or light brown pouches containing nicotine among other ingredients. They do not contain tobacco lead, dust, or stem. The nicotine may either be derived from tobacco plants or may be synthetic.

**OSU:	The Ohio State University**

**OSUWMC:	The Ohio State University Wexner Medical Center**

**PATH:	Population Assessment of Tobacco and Health**: a national, longitudinal study looking at tobacco use and health.

**PhenX Toolkit**:	Web-based catalog of high-priority measures related to complex diseases, phenotypic traits and environmental exposures. These measures were selected by working groups of experts using a consensus process. Use of PhenX measures facilitates combining data from a variety of studies, and makes it easy for investigators to expand a study design beyond the primary research focus.

**PHI**:	**Protected Health Information:** The HIPAA Privacy Rule provides federal protections for personal health information held by covered entities and gives patients an array of rights with respect to that information

**PII**:	**Personally Identifying Information:** Any representation of information that permits the identity of an individual to whom the information applies to be reasonably inferred by either direct or indirect means

**PI:	Principal Investigator**: is the lead scientist for a particular well-defined research project, such as a laboratory study or clinical trial.

**REDCap:	Research Electronic Data Capture** (web based software): is a secure, web-based application for building and managing online surveys and databases.

**RIT:	Department of Research Information Technology** at The Ohio State University.

**SAE:	Serious Adverse Event**: generally, any event which causes death, permanent damage, birth defects, or requires hospitalization is considered an SAE.

**TCORS**:	**Tobacco Centers of Regulatory Science**

**U.S.:	United States**

# 1. Study Summary

| **Study Title** | Human laboratory study of the effects of nicotine product claims on appeal, perceptions, and use behavior |
| --- | --- |
| **Study Design** | Laboratory Based Psychophysiological Study |
| **Primary Objective** | To examine how nicotine concentration and source claims affect engagement with oral nicotine pouch packaging |
| **Secondary Objective(s)** | To examine how nicotine concentration and source claims affect perceptions of oral nicotine pouches, intentions to try oral nicotine pouches, and product trial |
| **Research Intervention(s)/ Investigational Agent(s)** | N/A |
| **IND/IDE #** | N/A |
| **Study Population** | 150 young adult susceptible non-users and 300 adult tobacco users |
| **Sample Size** | 450 participants |
| **Study Duration for individual participants** | Approx 60 minutes |
| **Study Specific Abbreviations/ Definitions** | See Abbreviations |

# 2. Study Purpose & Aims

## 2.1 Aims & Expected Findings

**Aim 1:** **Examine the effects of nicotine concentration and source claims on engagement with ONP packaging using laboratory-based psychophysiological assessment.**

*H1a:* Higher nicotine concentration and “tobacco free” claims will increase visual and cognitive engagement with ONP packaging. *H1b:* These effects will be stronger among adult tobacco users than young adult susceptible non-users.

**Aim 2:** **Examine the effects of nicotine concentration and source claims on ONP perceptions, behavioral intentions, and ONP trial.**

*H2a:* Higher nicotine concentration and “tobacco free” claims will decrease perceived risks of ONPs, increase intentions to use ONPs, and increase ONP trial. *H2b:* These effects will be stronger among adult tobacco users than young adult susceptible non-users.

# 3. Background, Rationale, and Significance

## 3.1 Background

Oral nicotine pouches (ONPs) emerged in the U.S. in 2016, use is increasing, and sales are skyrocketing. ONPs are representative of a major shift by the tobacco industry to marketing non-tobacco nicotine products as alternatives to cigarettes, smokeless tobacco (ST), and even e-cigarettes (ECs). Nicotine is the critical driver of use and dependence, and the industry continuously manipulates ***nicotine dimensions*** to maximize appeal to consumers. Packaging is a primary marketing vehicle to increase product appeal, and ONP packaging features sleek designs, attractive colors, and prominently emphasizes nicotine ***concentration*** (e.g., 3 mg, 6 mg), and ***source*** ***claims*** (e.g., “tobacco free”) as a marketing tool. This project is part of the Ohio State University Tobacco Center of Regulatory Science U54 (OSU TCORS) and will address the public health impact of nicotine marketing using ONPs as an exemplar product to study the effects of packaging features communicating about nicotine in young adult susceptible non-tobacco users versus adult tobacco users.

In our preliminary studies, interest in ONPs was higher in tobacco users versus non-users, ONP packaging with higher nicotine concentration and “tobacco free” claims decreased perceived risks, and lower perceived risks were associated with ONP use. These preliminary findings highlight the significance of studying how ONP packaging features communicating nicotine dimensions that are manipulated by tobacco companies affect ONP appeal and trial in young adult non-users versus adult tobacco users.

Although ONPs may represent a harm reduction opportunity for adult tobacco users (e.g., smokers, ST users), ONP use by young people poses potential risks to public health. The goal of this project is to inform FDA regulations by examining how nicotine concentration and source claims on ONP packaging affect ONP appeal, product trial, and preferences for ONPs relative to ECs and other tobacco products among young adult susceptible non-users versus adult tobacco users.

## 3.2 Significance

**A1. How Novel Nicotine and Tobacco Products Appeal to Consumers.** The OSU-TCORS *integrative theme* is “***Flipping the Script: Using the Industry’s Nicotine Playbook to Maximize Public Health***.” The tobacco industry manipulates nicotine as a critical driver of tobacco use uptake and continued use. The industry has historically manipulated two nicotine dimensions, concentration and form, to create palatable “starter” products for new users and stronger “robust” products that facilitate transitions to regular use. More recently, with new, less expensive production technology the industry is using “synthetic” nicotine and marketing it as an appealing “tobacco-free” product. The OSU-TCORS is studying how the industry manipulates nicotine dimensions to inform potential Food and Drug Administration (FDA) regulations targeting nicotine for the protection of public health.

The OSU-TCORS’ research is guided by a consumer response model used by the industry to optimize the appeal of novel products, trial, and regular use (**Figure 1**).^1^ Consumers initially encounter novel products by engaging with marketing (i.e., packaging, advertising), and tobacco manufacturers carefully design marketing with branding, messaging, colors, and imagery to enhance visual and cognitive engagement with marketing, affect product perceptions and preferences, and increase use.^2^Unfettered marketing is a “*cause of the global spread of tobacco use and addiction.*”^3-5^ As U.S. policies increasingly restricted tobacco marketing channels (e.g., television, radio), manufacturers shifted to packaging as a primary marketing vehicle.^2^^,^ ^3^^,^ ^6^ Packaging features such as conveying constituents, product claims, and descriptors influence engagement with packaging, product appeal, and likelihood of use.^2^^,^ ^3^ Packaging is essential to tobacco marketing (e.g., point-of-sale power walls, advertising) and it is used to target specific groups, such as young people.^2^^,^ ^3^^,^ ^6^ Packaging regulations include requiring health warnings, prohibiting specific features (e.g., low, light, mild descriptors, tar/nicotine yields on cigarette packs) and restricting branding,^7^ and they can reduce tobacco use and associated morbidity and mortality.^8-10^ Studying packaging for novel products can inform regulations to improve public health.

**A2. This Project Will Advance Tobacco Regulation by Studying the Impact of Nicotine Marketing**

This project will advance tobacco regulatory science by studying how nicotine concentration and source claims on oral nicotine pouch (ONP) packaging affect ONP appeal, product trial, and preferences for ONPs relative to ECs and other products among young adult susceptible non-users versus adult tobacco users. This project focuses on ONPs because ONP sales are surging, interest and use are increasing, and they are exemplary of a major shift where tobacco companies are marketing non-tobacco nicotine products and prominently emphasizing nicotine concentration and source to appeal to consumers. Research on ONP packaging is scarce, but our preliminary studies indicate **nicotine concentration** and **source claims** on packaging increase the appeal of ONPs and highlight the significance of studying these packaging features as potential regulatory targets. This Project addresses two priority scientific domains of the Food and Drug Administration (FDA) Center for Tobacco Products (CTP), ***Marketing Influences*** and ***Behavior***, to inform potential regulations of packaging features for ONPs based on their effects in young adult susceptible non-users and adult tobacco users. This Project is highly integrated with the OSU-TCORS. It is guided by a common consumer response model, uses methods that tobacco companies use to optimize marketing,^11^^,^ ^12^ and it studies common nicotine dimensions, products, populations as other projects . This Project also leverages surveillance data on popular brands and features to ensure our approach is robust and externally valid. Together, the OSU-TCORS projects will provide comprehensive evidence to inform an overall regulatory strategy targeting industry manipulation of nicotine dimensions in ONPs, ECs, and other novel products.

## 3.3 Rationale

**B1. The Evolving U.S. Tobacco Marketplace and the Emergence of ONPs.** Over the past decade, tobacco companies have shifted their focus to non-tobacco nicotine products that are marketed as alternatives to cigarettes, smokeless tobacco (ST), and even e-cigarettes (ECs).^13^ ECs marked the start of this shift when they emerged in 2007 and brought tobacco harm reduction to the forefront because they are likely lower risk than cigarettes and other combustible tobacco.^13^ While some adult tobacco users switch to ECs or use ECs to quit, youth-targeted marketing, appealing features, and efficient nicotine delivery of modern ECs spurred a surge in EC use among young people.^14^ Now ECs are one of the most popular tobacco products^15^^,^ ^16^ and research is informing regulations to balance the impact of EC use in young non-users and adult tobacco users.^17-20^

ONPs recently entered the U.S. market–Zyn (**Figure 2- Figure removed for publication due to copyright**) and On! were introduced in 2016, ONPs were available nationally by 2019, and ONP brands have rapidly proliferated in the last 3 years (e.g., Velo, Rogue, Lucy, NIIN, Fre). ONPs come in containers of white sachets containing nicotine, flavorings, fillers, and other ingredients.^21^^,^ ^22^^,^^23^ They are placed between the cheek and gum to deliver nicotine, do not contain tobacco, do not require spitting, and some brands claim to use synthetic nicotine versus tobacco-derived nicotine.^23^ The most popular brands are owned by major tobacco companies (e.g., On! by Altria, Zyn by Swedish Match, Velo by RJ Reynolds, Rogue by Swisher), ONPs are priced similar to cigarettes and ST, and available in many flavors (e.g., mint, fruit, coffee).^23^

The nicotine concentration of ONPs ranges from 2 mg to >10 mg per pouch. Industry studies indicate ONPs deliver nicotine in similar ways to ST and nicotine lozenges.^24^^,^ ^25^ However, a CDC study suggests manufacturers are manipulating the pH, total nicotine, and free nicotine to alter ONP nicotine delivery, even within brands.^26^ This is consistent with tobacco companies’ past behavior in response to changes in the market. For example, as cigarette smoking declined in the 1990s, tobacco companies launched new ST products and manipulated nicotine to create “starter” products that were more palatable for young non-users to initiate and then “graduate” to “robust” products that are higher nicotine and more addictive.^27-29^ EC manufacturers followed this pattern, manipulating nicotine in ECs to optimize palatability and addictiveness.^14^ Now ONP manufacturers, many of which are major tobacco companies, also appear to be manipulating ***nicotine dimensions*** including concentration, source (i.e., isomer, tobacco derived or synthetic), and form (free-base nicotine content) to create products that appeal to young non-tobacco users and adult tobacco users.^26^ The industry’s history manipulating nicotine highlights the significance of studying ONPs at an early stage of their availability to inform regulations*.*

**B2. Appeal, Use, and Perceptions of ONPs.** ONPs have been available nationwide for 3 years and sales are increasing substantially, but research on ONPs is at a nascent stage. Sales of all oral nicotine products (e.g., pouches, gums, lozenges) at U.S. retail stores jumped from 0.1 million units in 2016 to 43 million units in 2019 and ONPs accounted for virtually all sales.^30^ From 2016 to 2020, ONP retail sales increased from 0.2 million to nearly 46 million units.^31^ The top-selling brands were Zyn, On!, Rogue, and Velo, all owned by major tobacco companies.^30^ Websites such as Nicokick also tout rapid sales growth, from 0.1 million units in 2019 to 4.8 million units in 2020.^32^ ONP sales surpassed snus during this period, and EC, cigarette, and snus sales all declined.^30^ Industry publications^33^ and ONP manufacturers^34^ promote this ONP sales success.

National survey data from 2019 and 2021 indicate about 1% of U.S. youth have used ONPs in the past 30 days.^15^^,^ ^35^ Data collected in 2021 by an ONP manufacturer indicate nearly 40% of youth aged 13-17 and 56% of young adults aged 18-20 were aware of ONPs, and they report that 1% and 4% used ONPs in the past 30 days, respectively.^36^ In a 2021 survey of young adults who never used ONPs, willingness to use ONPs was highest in dual users of non-combustible and combustible tobacco (44%), followed by exclusive non-combustible users (34%), exclusive combustible users (29%), and non-users (15%).^37^ Nearly half (49%) were unsure if ONPs are less harmful than cigarettes and risk perceptions did not differ by tobacco user status.^37^ Research with U.S. adults indicates awareness and use of ONPs is highest among adult tobacco users, especially non-combustible tobacco (e.g., ST) users and dual/poly users, but this evidence is limited to two studies.^38^^,^ ^39^

Overall, evidence indicates appeal and use of ONPs is highest among adult tobacco users and ONPs are gaining appeal in young adult non-users.^37^ For comparison, in the few years after ECs became available nationally, EC use was highest in adult cigarette smokers, but appeal and use steadily increased, including among young people.^40^ By 2015, high nicotine concentration, nicotine salt-based ECs accounted for the majority of EC sales, marketing was pervasive, and ECs became – and remain – one of the most popular tobacco products in the U.S.^14-16^^,^ ^41^^,^ ^42^ Research on the appeal and use of ONPs is at an early stage, but the available evidence indicates awareness, appeal, and use is similar to ECs early in their availability in the U.S*.*

**B3. ONP Packaging Prominently Emphasizes Nicotine Dimensions to Enhance Appeal.** ONP marketing relies on strategies the tobacco industry has used to increase use of their products^43-50^ and promotes novel features to appeal to consumers. Marketing content for ONPs features claims they can be used anywhere, are an alternative to cigarettes, ST, and ECs, and are “spit free” and “smoke free.”^51^ Manufacturers co-promote ONPs and cigarettes (e.g., On! with Marlboro) with messaging that ONPs can be used to circumvent policies prohibiting smoking.^52^ Like ECs,^53^ ONPs are promoted by celebrities and influencers on social media,^54^ tactics that contributed to the surge of EC use in young people.^41^

Two unique features of ONP marketing prominently stand out: ***nicotine concentration*** and ***source claims*** (**Figure 3 – removed for publication due to copyright**). As of 2022, all ONP brands featured nicotine concentration on packaging^32^ and this nicotine dimension is used in marketing to appeal to tobacco users and non-users. For example, Nicokick.com indicates: “*the [nicotine] strength you pick will depend on a number of factors”* including “*whether you are a first-timer or you’ve used nicotine for years.”**^55^* Zyn ads display packaging to emphasize 3 mg ONPs provide “*fresh nicotine satisfaction”* and 6 mg ONPs deliver “*even more nicotine enjoyment.*”^56^ Marketing surveillance also shows that most ONPs use nicotine source claims, including “tobacco free” or “synthetic” nicotine in marketing.^51^^,^ ^57^^,^ ^58^ Retailers leverage this to appeal to consumers as well, emphasizing ONPs have “*no smoke,”* are “*discreet*,*” “convenient,*” and *“can easily fit your nicotine kick into your daily routine”**^59^* without smoking, spitting, or vaping. This is a stark contrast to traditional ST marketing, for example, which emphasizes ruggedness, masculinity, and outdoors.^60^^,^ ^61^

The appeal, uptake, and use of ONPs will be impacted by how consumers engage visually and cognitively with ONP packaging, and how packaging features affect ONP perceptions and preferences.^1^ Information about nicotine concentration can promote tobacco use by affecting perceived risks.^62-68^ Research on tobacco^69-75^ and other consumer goods (e.g., foods)^76^^,^ ^77^ shows that claims like “tobacco free” reduce perceived risks, increase beliefs that products are “healthy,” and increase consumption. Though research on ONPs is limited, recent evidence suggests few young adults understand “tobacco free” nicotine source claims for ONPs and ECs,^78^ and “tobacco free” claims decrease perceived risks and increase intentions to use ECs.^79^ Overall, research supports the hypothesis that nicotine concentration and source claims on ONP packaging can affect the appeal of ONPs, perceptions, and ONP use.

**B4. Packaging Is a Central Component of Tobacco Regulations to Promote Public Health.** The 2009 Tobacco Control Act authorized the FDA to regulate the marketing, distribution, and sale of cigarettes, smokeless, and roll-your-own tobacco.^80^ The 2016 Deeming Rule expanded this regulatory authority to all products containing or derived from tobacco, and 2022 legislation (H.R. 2471) further expanded FDA’s authority to include synthetic nicotine products.^81^ These laws recognize the importance of packaging as a tobacco regulatory target by implementing initial packaging regulations and granting FDA the authority to enact additional packaging regulations if evidence demonstrates they are appropriate for the protection of public health.^80^ Thus, packaging regulations must be guided by research on their impact in tobacco users and non-users.^80^^,^ ^82^

FDA has identified packing research as a tobacco regulatory science priority,^58^ and this project will provide evidence to inform regulations for ONPs and other nicotine products in two important ways. First, the Tobacco Control Act authorizes FDA to regulate quantitative information about nicotine and other constituents on packaging. Cigarette companies historically included nicotine and tar yields (e.g., Marlboro Lights, “1.3 mg nicotine per cigarette”) on packaging and other marketing as a strategy to increase the appeal of smoking by reducing perceived risks.^68^^,^ ^83^ NCI Monograph 13 concluded that quantitative information about nicotine and tar yields on cigarette packs misled consumers and fostered continued smoking,^68^ and The Framework Convention for Tobacco Control recommends prohibiting their use.^84^ Our preliminary studies indicate nicotine concentration on ONP packaging may similarly affect perceived risks, and this Project will provide evidence to inform FDA regulations in this area.

Second, the Tobacco Control Act prohibits use of specific descriptors (low, light, mild)^68^ and authorizes FDA to regulate use of implicit or explicit claims based on how they affect consumers’ perceptions of risk.^80^^,^ ^82^^,^ ^83^ Claims like “tobacco free” used on ONP packaging have been shown to mislead consumers about the potential risks of tobacco products.^69-75^ Our preliminary studies indicate nicotine source claims shift ONP perceptions in a way that promotes ONP use. FDA has taken action to restrict similar claims, and for novel products like ONPs evidence on their effects can inform potential regulations surrounding their use, and it can be used in the review of premarket applications (e.g., Zyn, On!, Rogue, and Lucy have submitted ONP premarket applications), and future modified risk tobacco product applications. This project will generate timely evidence on the effects of nicotine concentration and source claims on ONP packaging to inform potential regulations*.*

## 3.4 Innovation

This project advances tobacco regulatory research and is highly innovative for several important reasons:

(1) There is little research on how ONP packaging features influence the appeal, perceptions, and use of ONPs. This Project will provide timely evidence on the public health impact of ONP packaging features emphasizing dimensions of nicotine on these outcomes.

(2) This Project uses an innovative approach combining laboratory-based physiological measures of visual and cognitive engagement with ONP packaging, self-report measures of ONP perceptions and use intentions, and a behavioral lapse task capturing ONP trial. These are robust methods that have been used independently to study tobacco packaging, but our use of them in combination to study ONP packaging is highly innovative.^12^^,^ ^85^

(3) Our approach is highly innovative and it aligns with the OSU-TCORS integrative theme “***Flipping the Script***” because we leverage methods that commercial marketers – including tobacco companies – use to optimize marketing to promote their products, such as psychophysiological assessment.^11^^,^ ^12^

# 4. Study Design & Measures

## 4.1 Research Design Overview

This OSU-TCORS Project includes two complementary studies. **Study 1** (**Aims 1** and **2**) is a 2 (young adult susceptible non-user/adult tobacco user) x 3 (labelled nicotine concentration: none, low, high) x 3 (source claim: none, “tobacco free nicotine,” “synthetic nicotine”) laboratory experiment to examine how nicotine concentration and source claims affect engagement with ONP packaging using psychophysiological measures, self-reported ONP perceptions and use intentions, and a lapse task assessing ONP trial. **Study 2** is an online DCE to assess nicotine concentration and source claim effects on ONP preferences relative to ECs, cigarettes, ST, and other products. This protocol encompasses procedures for Study 1. Study 2 will be submitted as a separate IRB application. **Figure 5** shows how our approach aligns with the Tobacco Consumer Response Model that guides the OSU-TCORS,^1^ and the theories of communication and behavior^86-92^ that inform our approach and outcomes. According to these models, ONP packaging will influence behavioral outcomes if consumers engage with packaging features and information is encoded into memory, outcomes captured in Study 1.^92^^,^ ^93^ ONP packaging features can influence perceived risks (Studies 1 and 2), preferences for ONPs (Study 2), intentions to use ONPs (Studies 1 and 2), and ONP trial (Study 1), established predictors of future use.^86^^,^ ^87^^,^ ^90^^,^ ^91^ Together, Studies 1 and 2 capture outcomes that are critical to understand the impact of ONP packaging features^89^^,^ ^93^ using methods reflecting two primary contexts in which consumers engage with packaging: when they interact with packaging directly (Study 1) and when they encounter packaging for multiple products at the point of sale (Study 2).^1^^,^ ^2^^,^ ^6^ Our outcomes can inform tobacco regulation because they are based on valid, rigorous measures of consumer response to packaging.^11^^,^ ^12^^,^ ^85^^,^ ^94^ Studies 1 and 2 use inputs from surveillance data on sales to maximize external validity by anchoring procedures to ONPs and other products available in the U.S. market.

## 4.2 Experimental Procedures

### Psychophysiological

Study 1 procedures are based on our preliminary studies and our other prior work.^95-99^ Visits will occur in a private room at the OSU CTR. Participants will complete pre-exposure self-report measures (described in **4.4**) and then will be randomly assigned to conditions in a 3 (**Nicotine Concentration**: None on label, Low, High) x 3 (**Nicotine Source Claim:** None, “Tobacco Free,” “Synthetic Nicotine”) between-subjects design. We will stratify randomization by young adult susceptible non-users and adult tobacco users for balance across the conditions.

Participants will be seated in front of a high-resolution monitor at a comfortable viewing distance (24–32 inches) and they will have 4 pre-gelled electrocardiogram (ECG) and two skin conductance (electrodermal activity; EDA) sensors affixed to their forearms and fingers to collect ECG and EDA data. We will conduct 3 calibration tests to assure eye-tracking data quality.

For the nicotine concentration and nicotine source claim condition to which they are randomized, participants will view 16 ONP pack images in random order (**Figure 7**). In each condition, pack images will vary within-subjects by 2 features: Brand (n=4 brands, e.g., Zyn, Velo, On!, Rogue) and flavor (n=4 flavors, e.g., mint, fruit, coffee, unflavored) (**Figure 7**). All pack images will display FDA’s required nicotine addiction warning. **Figure 6 (Figure 6 - removed for publication due to copyright)** shows example images. Using market surveillance data, we will identify the 4 most popular ONP brands, confirm content and placement for nicotine concentration and source claims, and identify the 4 most popular flavors by brand. This will maximize external validity of our stimuli and enable us to account for brand and flavor in the design, control for these as needed in analyses, and conduct exploratory analyses of their effects. We will digitally edit pack images to be consistent across conditions as shown in the examples in **Figure 6 (Figure 6 - removed for publication due to copyright)**. In conditions with no nicotine concentration/source claims, labels will include visual/content controls (e.g., similarly sized slogans, branding) in the same location on the pack image to ensure valid comparisons between conditions.

Participants will view each pack image for 10 s, which provides adequate time to capture psychophysiological responses.^98^ After each ONP pack image, participants will view images of non-tobacco/non-nicotine products with similar features, such as alcoholic and non-alcoholic beverages to serve as a “washout” between the ONP pack images. The order of non-tobacco/non-nicotine product images will also be randomized to prevent any potential ordering effects in the design. Between each image, participants will view a blank screen with the words “Get Ready” for 5 s to return psychophysiological measures to baseline.^98^ The total time viewing stimuli will be approximately 10 min. After the exposure, participants will complete self-report measures of ONP perceptions, use intentions, and a task assessing ONP trial (self-report measures and the lapse task are described below). At the conclusion of the visit, all participants will be debriefed and given information on the risks of tobacco and nicotine use and information on free, evidence-based cessation resources.

### Lapse Task

After completing post-exposure self-report measures, participants will watch a brief distractor video (e.g., about nature) and will complete a lapse task assessing ONP trial. This is a valid behavioral measure that was originally developed for smoking cue reactivity research and used in prior work by others^12^^,^ ^100^^,^ ^101^ and our team.^102^ In the visit room, participants will be presented with ONP packages corresponding to the nicotine concentration by nicotine source claim condition to which they were randomized and the brands and flavors shown in the stimuli.

We will use participant deception in two components of the lapse task. The lapse task session length will be 25 minutes. We will instruct participants that they can choose to try the product at any time, but they can earn $0.20 for every 1 minute they delay trying totaling to $5 if they delay/abstain for the full time. Participants will be instructed that the potential to continue to earn money will be eliminated at the time if/when they attempt to use any of the ONPs.^100^^,^ ^101^ When participants attempt to use any of the ONPs, research staff will stop the task and record the outcome as a trial, and which product they opened. For participants who do not attempt to try the ONPs for the entire 25 minutes, the outcome will be recorded as a non-trial. This will be deception (deception component 1) since we will pay all participants $5 regardless of whether they attempt to try the ONPs. Deception for this part is necessary to provide participants an incentive structure to abstain from using the ONPs. It does not create any increased risk to participants.

Participants will be instructed that they are required to be in the room for the full 25 minutes regardless of trial to prevent participants from intentionally trying the ONPs for the purpose of leaving early. Participants will not be allowed to use their phones or other personal devices during this time and must stay awake for the 25 minutes. Staff will monitor participants from a separate control room, stop participants at the first attempt to try ONPs, and as noted above will record the time to first attempt and the ONP product they selected including the brand and flavor. At that time or at the session conclusion, whichever is first, we will inform participants they will receive the full $5 regardless of trial. The package **will not** contain ONPs (deception component 2); we will substitute the ONPs with commercially available pouches that contain food grade ingredients (i.e., sugar) and do not contain nicotine or tobacco. Deception is necessary in this step to assess ONP trial without the potential risk of exposure to tobacco or nicotine.

### Debriefing

At the conclusion of the lapse task, all participants will be debriefed. We will provide a prompt opportunity for participants to obtain appropriate information about all study deception elements in the lapse task and will explain the rationale for the deception. All participants will be informed about study deception elements including $5 payment regardless of trial and that the pouches in the lapse task containers are not ONPs.

All participants will be provided information about the potential risks of nicotine products, and tobacco users will also be provided information on cessation resources.

## 4.3 Measures

All measures use rigorous, valid assessments based on our team’s previous work and align with our conceptual rationale (**Figure 5**). Self-report measures will be collected using REDCap and will be aligned with the PhenX Toolkit^103-105^ where possible to maximize reproducibility and data harmonization with other TCORS. Stimuli presentation and psychophysiological measures will be collected using the iMotions platform.

**Pre-Exposure.** Participants will complete measures of demographics (sex, gender, age, race/ethnicity, education, employment status, household income, subjective financial situation, family socioeconomic status),^16^^,^ ^103-106^ subjective numeracy,^107^ BRIEF health literacy,^108^ beliefs about tobacco and nicotine and tobacco and nicotine product use (duration, frequency, products used, usual brands, dependence).^103-105^ We will also assess ONP awareness and marketing exposure^105^ as in our preliminary studies**.**

**Visual Attention.** Visual attention will be measured via eye movements using SmartEye Aurora eye tracker with a 60 Hz sampling rate. Areas of interest (AOIs) will be predefined for each pack image including: 1) Full pack image; 2) Nicotine concentration; 3) Nicotine source claim; 4) Branding (i.e., brand name, flavors); 5) Warning label. The primary outcomes for visual attention will be dwell time, time to first fixation, and number of fixations (i.e., saccades) to AOIs.^109-111^

**Cognitive Attention.** Conceptualized as the cognitive resources allocated to encode a message into working memory, attention will be operationalized as the difference in heart rate (HR) between a baseline period prior to each pack image and during each second of the pack image exposure^98^^,^ ^112-114^ assessed with ECG. Beats per minute (BPM) will be collected with the Shimmer3 sampled at 512 Hz. This is an established psychophysiological measure of cognitive resources devoted to stimuli processing.^98^^,^ ^112-114^

**Arousal.** Arousal is an indicator of physiological activation of the sympathetic nervous system and will be measured by skin conductance using Shimmer3 Galvanic Skin Response EDA sensors sampled at 128 Hz.^98^

**Recall.** We will capture unaided recall, a measure of storage in memory,^93^ of nicotine concentration and source information corresponding to conditions to which participants are randomized with open ended questions. Responses will be coded, and coding reliability will be established, to create a measure of recall of information about nicotine dimensions in the stimuli.^97^^,^ ^115^

**ONP Perceptions.** We will capture perceptions of ONPs with 7 items that align with expert recommendations for assessing cognitive and affective aspects of tobacco risk perceptions.^116^ Items will assess perceived risk of health harm from ONPs, perceived risk of addiction from ONPs, worry about harm, and worry about addiction.^117-120^ The items are on a 1–7 response scale, have excellent reliability, and are averaged to create an overall score. We will also capture perceived harm and addictiveness of ONPs relative to cigarettes, ST, and ECs using valid items.^115^^,^ ^121^^,^ ^122^

**ONP Intentions.** We will assess curiosity, interest, and likelihood of using ONPs post-exposure. These are valid predictors of future use of novel tobacco and nicotine products among never users.^118^^,^ ^120^^,^ ^123^ Items are on a 1–7 scale, have excellent reliability, and are combined to create an overall ONP intentions score.

**ONP Trial (Lapse Task).** This is a valid behavioral measure that was originally developed for smoking cue reactivity research and used in prior work by others^12^^,^ ^100^^,^ ^101^ and our team.^102^ The lapse task will assess ONP trial, time to trial, and ONP product selection. For additional details, see section 4.2.

## 4.4 Subject Compensation

Participants will receive up to $105 for completing procedures; those who fail eye tracking calibration after 3 attempts will receive $25 but will not complete subsequent procedures. Participants who complete all assessments will receive $105.

# 5. Subject Selection

## 5.1 Inclusion & Exclusion

**Inclusion: Participants must meet criteria for either A. or B. All must meet criteria for C.**

A. **Young adult susceptible non-users*** **aged 18–24** who have never used ONPs and have never used but are susceptible to using either
   a. **combustible tobacco only** (cigarettes, cigars, waterpipe)
   b. **non-combustible tobacco only** (ST, ECs, heated tobacco products)
   c. **combustible and non-combustible tobacco** (i.e., dual susceptibility)

*For young adult non-users, we will capture susceptibility, a robust predictor of future tobacco use in young adults,^124-127^ at screening using valid items.^128^

B. **Adult tobacco users** aged 18–65** who have never used ONPs OR have used ONPs >3 months ago and 10 times or less in their lifetime. All adult tobacco users must also be:
   a. **exclusive combustible tobacco users,**
   b. **exclusive non-combustible tobacco users**,
   c. **dual users**.

**For adult tobacco users, we will define current use as use of combustible and/or non-combustible tobacco every day or some days for 6 months or longer.

C. Willing and able to complete an in-person lab visit.

**Exclusion**

A. Age <18 or >65
B. Are not susceptible non-users or are not current tobacco users as defined in inclusion A and B.
C. Are unwilling or unable to complete and in person lab visit.
D. Have used ONPs within the past 3 months
E. Have used ONPs more than 10 times in their lifetime

## 5.2 Justification for Inclusion & Exclusion

**Why focus on young adult susceptible non-users and adult tobacco users?** This project focuses on these groups because they align with the public health standard guiding FDA tobacco regulations. These are groups where ONP marketing can impact public health by driving at risk non-users to initiate ONPs and established tobacco users to switch products or quit. Although This Project is not designed to assess these behavioral outcomes longitudinally, we will use robust methods to capture the immediate impact of ONP marketing in these groups to provide evidence that is complementary to the other OSU-TCORS projects. Together, findings the OSU-TCORS projects will deliver comprehensive evidence that can inform FDA regulations targeting nicotine dimensions.

Our upper age limit is 65 because novel nicotine/tobacco product use is rare in adults older than 65.^129^^,^ ^130^ Given the tobacco industry’s history targeting younger adults with marketing,^131^^,^ ^132^ higher appeal of novel products in younger adults,^129^^,^ ^130^^,^ ^133^ and evidence that ONPs appeal to younger adults,^37^ we will use recruitment quotas to achieve equal numbers of younger (18–24) and older (25–65) adults by tobacco user group. This also aligns our approach with the public health standard for FDA tobacco regulations.^80^^,^ ^82^

Given the increase in ONP exposure, the population of completely naive participants is diminishing. We revised our ONP eligibility criteria to account for this and will control for prior use in our analysis. Modifying the criteria will also likely improve the representativeness of certain demographic groups in our sample (e.g., males) because they are more likely to use oral/smokeless tobacco products.

## 5.3 Recruitment

For recruitment, we will primarily use: 1) social media advertising; 2) online classifieds; 3) community-based advertising; 4) word of mouth referrals; 5) re-contacting participants from prior related tobacco studies who have agreed to be recontacted; and 6) obtaining a list of OSU students stratified by age from the OSU Registrar Office. We will ask for students who are 18 -25 years and/or >25 years old to supplement other recruitment methods and help fill quotas where needed. The investigators will oversee recruitment in collaboration with the OSUCCC Social Media team.

## 5.4 Number of Participants

Our total target sample is 450 participants, including a total of 150 young adult susceptible non-users and 300 adult tobacco users. For young adult susceptible non-users ages 18 to 24, we will recruit approximately 50 participants who are susceptible to combustible tobacco only, susceptible to non-combustible tobacco only, or susceptible multiple tobacco products (i.e., dual or poly susceptibility). For young adult tobacco users ages 18-24 years, we will recruit approximately 50 participants who exclusively use combustible tobacco, who exclusively use non-combustible tobacco, or who use multiple tobacco products (i.e., dual or poly use). For adult tobacco users ages 25 to 65, we will recruit approximately 50 participants who exclusively use combustible tobacco, who exclusively use non-combustible tobacco, or who use multiple tobacco products (i.e., dual or poly use). However, our overall number of participants we will recruit will include 150 young adult susceptible non-users and 300 adult tobacco users as reflected in the table below.

| **Table 1. Study 1 Overall Sample Size and Approximate Subgroups of Participants** | | | | |
| --- | --- | --- | --- | --- |
|  | **Exclusive Combustible** | **Exclusive Non-Combustible** | **Dual** | **Total** |
| Susceptible Non-Users 18-24 years | 50 | 50 | 50 | **150** |
| Current Users 18-24 years | 50 | 50 | 50 | **150** |
| Current Users 25-65 years | 50 | 50 | 50 | **150** |

## 5.5 Feasibility

We have extensive experience using psychophysiological measures including eye tracking, heart rate, and galvanic skin response as measures of engagement with tobacco packaging, marketing, and messaging.^95-99^^,^ ^134-138^ These measures capture visual and cognitive engagement with stimuli in the moment of exposure by tapping into biological processes indicative of attention, arousal, cognitive processing, and appetitive or aversive reactions that are not accessible by self-report.^11^^,^ ^113^^,^ ^139^ They are highly significant to tobacco regulatory science because they provide objective measures of tobacco marketing influences.^11^^,^ ^85^

Dr. **Mays** used psychophysiological methods to identify optimal tobacco public education message features in young adult tobacco users (n=59) and susceptible non-users (n=61).^98^^,^ ^140^ Testing an initial larger set of messages, we used the combination of eye tracking, heart rate, skin conductance, and other measures to identify message features that increased cognitive engagement, maximized encoding, and minimized defensive processing.^98^^,^ ^136^^,^ ^140^ We showed in a subsequent trial the optimized messages significantly reduced tobacco initiation among susceptible non-users and reduced use in tobacco users at higher doses of exposure.^141^

Dr. **Klein** examined young adult tobacco users’ visual engagement with parody warnings by EC manufacturers versus FDA warnings in EC ads.^95^ Participants viewed parody warnings longer than FDA warnings, and branding features (e.g., people, logos) drew attention away from warnings overall.

Dr. **Klein** has also examined engagement with tobacco marketing and packaging and how regulations such as health warning labels affect engagement and the appeal of tobacco products. One study demonstrated that smokeless tobacco users primarily attended to packaging, slogans, and logos in smokeless tobacco ads, but graphic health warnings in ads attracted more attention and produced greater recall than text-only warnings.^96^ Another study examined adult cigarette smokers’ engagement with branding in Natural American Spirit ads with graphic or text only health warnings.^97^ Smokers spent less time viewing cigarette packages and claims (“Natural,” “Tastes Better”) in ads with graphic warnings versus the text warnings. Visual attention was greater for graphic versus the text warnings, and attention to the graphic warnings mediated the difference in post-exposure recall between the graphic and text only warnings.^97^

Our experience using psychophysiological methods to study tobacco marketing, packaging, and messaging supports: (1) The feasibility of conducting Study 1 with a local sample of young adult susceptible non-users and adult tobacco users; and (2) The ability of psychophysiological measures to detect differences in engagement with these stimuli between tobacco users and susceptible non-users*.*

# 6. Study Procedures

## 6.1 Eligibility Screening

Recruitment advertising will direct interested participants to a landing page with information about the study and a link to complete an online eligibility screener in REDCap. Study staff will review responses in REDCap and contact respondents about next steps. Information collected in the eligibility screener will only be used to confirm eligibility and to communicate with participants; for example: to verify eligibility should any eligibility responses need clarification and to schedule a lab appointment.

## 6.2 Consent Process

Eligible participants will be contacted by phone, email, or text message to discuss the study, answer and questions, and schedule a lab appointment. At the start of the lab appointment, participants will complete study enrollment by providing electronic informed consent via REDCap with the study coordinator present to answer any questions. The informed consent will be a written explanation of the study, including a description of the nature, purpose, risks, and any potential benefits. The informed consent form will follow OSU IRB institutional consent guidelines to include clear written instructions to participants to guide the consent process, and our research staff will also be available to discuss the study and clarify any questions that potential participants may have. The voluntary and confidential nature of the study, limits to confidentiality, and the participant’s right to withdraw at any time will be emphasized. A copy of the informed consent will be available to the participant via email from REDCap. Participant signatures will be obtained using a: typed full legal name, date, and electronic signature (via mouse or finger if using a touchscreen device, such as a mobile device).

## 6.3 Subject Registration

Study registration will occur after the informed consent process is completed and the electronic consent form is signed and initial eligibility screen is confirmed. No study procedures will be conducted until after the consent form is signed. Enrollment is confirmed once consent is received by research staff and will be registered in REDCap.

## 6.4 Experimental Study

After the subject is enrolled, the participant may proceed with the study and follow procedures set forth in section 4.2.

# 7. Data Collection and Management

## 7.1 Safeguards for subject confidentiality

Self-report data from eligibility screening, consent, and the brief survey will be collected and stored in REDCap, a secure electronic management platform for research study tracking, data entry, collection, and management. REDCap is hosted and maintained by The Ohio State University. Identifiers collected during screening and enrollment will include participants name, email address, telephone number, and mailing address. This information will be used to verify eligibility, communicate with participants, and issue participant incentive payments at study completion. The study REDCap database will only be accessible to trained, authorized research team members designated by the Principal Investigator through password-protected university computers. Downloaded data files (i.e., backups and final files at the conclusion of data collection) will be securely stored on a secure network drive that is accessible only via password-protected university computers behind the university firewall by research personnel designated by the Principal Investigator.

Psychophysiological measures including eye tracking, heart rate, and galvanic skin response, will be completed using iMotions software and equipment during an in-person lab visit. Eye tracking will be measured using SmartEye Aurora device. Heart rate and galvanic skin response will be measured using two Shimmer 3 devices. Psychophysiological data will be collected and stored in a secure network. Psychophysiological data will be initially collected on a dedicated lab laptop that has been approved for this purpose by OSUWMC IT. Data will be stored on an X: drive on the laptop that has been set up by OSUWMC IT and transferred to a secure network drive that is accessible only via password-protected university computers behind the university firewall by research personnel designated by the Principal Investigator. Psychophysiological data will not contain any identifiers. No identifiers will be stored in iMotions nor on the computer that houses iMotions. Only subject number will be used for psychophysiological data identification.

## 7.2 Record Retention

All research-related records will be maintained for at least 3 years after the research has ended. The primary research data will be retained at Ohio State for a minimum of 5 years after final project closeout.

# 8. Risks and Benefits

## 8.1 Risks and Benefits

This study involves no more than minimal risk. It does not involve drugs, devices, or medical procedures. PHI is not collected during this study.

The primary potential source of risks in this study is the social risk of gathering behavioral information about tobacco use and tobacco-related attitudes, perceptions, and beliefs. These potential risks include fear of disclosure of potentially sensitive information about tobacco use. Some people may feel uncomfortable answering questions about their tobacco use history or smoking or smokeless tobacco use status. Informed consent will be obtained from all participants prior to study enrollment, emphasizing the voluntary nature of the study.

There are no direct benefits to participants in this study; however, the findings of this study will be applied to inform future research and intervention efforts designed to reduce the public health burden of tobacco use in the United States.

## 8.2 Minimizing Risks and Benefits

Eligibility screening, informed consent, and electronic study tracking will be securely maintained and entered in REDCap. REDCap is a secure electronic management platform for research study tracking, data entry, collection, and management. REDCap is hosted and maintained by The Ohio State University. It is designed for the specific purpose of data collection for research studies, providing: 1) an intuitive interface for validated data entry; 2) audit trails for tracking, data manipulation, and export procedures; 3) automated export procedures for seamless data downloads to common statistical packages; and 4) procedures for importing data from external sources. The REDCap application and databases are secured on The Ohio State University’s server with firewall, backup, and environmental and user-level controls. REDCap is only accessible through secure, password-protected computers within locked offices at the Center for Tobacco Research or via VPN. Validation will also be required at the user level, where only authorized study team members have access to the project databases within REDCap based on their OSU ID. Access to all study data will be restricted to the study MPIs and fully trained, authorized members of the research team.

## 8.3 Risk Justification

Evidence on the appeal, perceptions, and use of ONPs is at an early stage, and much of the available evidence is from studies by ONP manufacturers. There is no published research on how ONP packaging features communicating dimensions of nicotine affect the appeal of ONPs in young non-users and adult tobacco users to inform regulations. This Project addresses these research gaps and is highly significant to tobacco regulatory science for several reasons.

(1) ONPs are a novel product, exemplary of a shift in the U.S. to non-tobacco nicotine products marketed emphasizing industry-manipulated nicotine dimensions to appeal to consumers. Evidence suggests the appeal and use of ONPs are increasing like ECs shortly after their introduction, and rigorous research on the appeal, perceptions, and use of ONPs early in their availability is needed to inform regulations.

(2) Packaging is a primary tobacco marketing vehicle, and nicotine concentration and source claims are pervasive on ONP packaging. This Project will rigorously examine the effects of ONP packaging features emphasizing ***nicotine concentration*** and ***source*** in young adult susceptible non-users versus adult tobacco users to understand their public health impact and inform potential regulations for ONPs and other products emphasizing nicotine dimensions in their marketing.

(3) This Project addresses FDA tobacco regulatory scientific domains ***Marketing Influences*** and ***Behavior,*** and it includes FDA priority populations (e.g., young adults) to characterize the public health effects of ONP packaging features.

# 9. Adverse Event Reporting

Dr. Mays is responsible for reporting unexpected problematic events involving any aspect of the study to The Ohio State University IRB according to institutional guidelines. Unanticipated problems to be assessed include adverse events, deviations from the study design or protocol, problems with informed consent, and confidentiality violations. Participants will be encouraged to contact Dr. Mays at any time during the study.

AEs and SAEs will be recorded as described in the Adverse Event SOP. We expect study related AEs and SAEs to be rare and, therefore, no formal statistical analysis of the occurrence or rate of AEs and SAEs is planned.

Reportable serious and related adverse events will be reported by telephone or email to the IRB within the 5 days of our receipt of information regarding the event and written reports will be submitted within 10 days. Subjects will be informed of any changes in risk.

# 10. Statistical Considerations and Data Analysis

## 10.1 Power and Sample Size

Our analyses for Study 1 will examine the effects of nicotine concentration and source claims on engagement with ONP packaging based on psychophysiological measures (**Aim 1**) and their effects on post-exposure measures of ONP perceptions, behavioral intentions, and ONP trial (**Aim 2**). In both aims, we will examine if nicotine concentration and source claims differentially affect young adult susceptible non-users and adult tobacco users. Our target sample is 450 participants, including 150 young adult susceptible non-users and 300 adult tobacco users. For a 3 (**Nicotine Concentration**: None on label, Low, High) x 3 (**Nicotine Source Claim**: None, “Tobacco Free,” “Synthetic Nicotine”) between-subjects design with 16 repeated measures, *α* = .05, and *r* among repeated measures of .5, this sample provides >80% power to detect small (*f* = .11) mean differences between levels for nicotine concentration or source claim by tobacco user status. For **Aim 1**, these effect sizes are consistent with our prior work^98^ and psychophysiological research generally.^92^ For **Aim 2**, our sample for Study 1 positions us to detect effects similar to our prior work examining how features of tobacco marketing (e.g., advertising, packaging) affect risk perceptions, behavioral intentions, and other outcomes.

## 10.2 Statistical Analyses

Visual attention data will be analyzed to assess differences in attention overall (dwell time) and viewing patterns to specific AOIs (e.g., time to first fixation for nicotine concentration and source claims). Psychophysiological data will be pre-processed and cleaned according to published standards.^98^^,^ ^110^^,^ ^111^ For each exposure, we will calculate the change in heart rate (attention) and skin conductance (arousal) by subtracting the last second of baseline data from each second of viewing time collected during the exposure. All outcomes (visual attention, heart rate, skin conductance, etc.) are continuous and thus we will use a generalized estimating equation (GEE) approach to analyze differences in means between conditions (both nicotine concentration and nicotine source claim). This approach is analogous to a two-way ANOVA with repeated measures but allows for additional covariates and additional robustness in modeling the correlation of observations within individual participants. In all analyses we will examine interactions by condition and susceptible non-user/tobacco user status to test *H1b* that effects of condition are stronger among adult users*.* We will also conduct exploratory analyses to examine if the effect of condition varies by sex, race, and ethnicity, although we do not expect this based on our prior work. Analyses for **Aim 2** are identical except for changing the outcome measure, as the interest again is in differences in means (e.g., differences in perceived risk, time to ONP trial by condition).

# 11. References

1.	Rees VW, Kreslake JM, Cummings KM, et al. Assessing consumer responses to potential reduced-exposure tobacco products: a review of tobacco industry and independent research methods. Cancer Epidemiol Biomarkers Prev. 2009;18(12):3225-40.

2.	National Cancer Institute. The role of the media in promoting and reducing tobacco use. In: U.S. Department of Health and Human Services, editor. Bethesda, MD2008.

3.	Henriksen L. Comprehensive tobacco marketing restrictions: promotion, packaging, price and place. Tob Control. 2012;21(2):147-53.

4.	Health NCfCDPaHPUOoSa. Preventing Tobacco Use Among Youth and Young Adults: A Report of the Surgeon General. 2012.

5.	US Centers for Disease Control and Prevention Office on Smoking and Health. The Health Consequences of Smoking—50 Years of Progress: A Report of the Surgeon General. 2014.

6.	Wakefield M, Morley C, Horan JK, Cummings KM. The cigarette pack as image: new evidence from tobacco industry documents. Tob Control. 2002;11 Suppl 1:I73-80.

7.	Smith CN, Kraemer JD, Johnson AC, Mays D. Plain packaging of cigarettes: do we have sufficient evidence? Risk Manag Healthc Policy. 2015;8:21-30.

8.	Levy DT, Ellis JA, Mays D, Huang AT. Smoking-related deaths averted due to three years of policy progress. Bull World Health Organ. 2013;91(7):509-18.

9.	Levy DT, Mays D, Yuan Z, Hammond D, Thrasher JF. Public health benefits from pictorial health warnings on US cigarette packs: a SimSmoke simulation. Tob Control. 2017;26(6):649-55.

10.	Levy DT, Yuan Z, Luo Y, Mays D. Seven years of progress in tobacco control: an evaluation of the effect of nations meeting the highest level MPOWER measures between 2007 and 2014. Tob Control. 2018;27(1):50-7.

11.	Meernik C, Jarman K, Wright ST, et al. Eye Tracking Outcomes in Tobacco Control Regulation and Communication: A Systematic Review. Tob Regul Sci. 2016;2(4):377-403.

12.	Robinson JD, Drobes DJ, Brandon TH, Wetter DW, Cinciripini PM. Evaluating Point of Sale Tobacco Marketing Using Behavioral Laboratory Methods. Tob Regul Sci. 2016;2(4):414-25.

13.	Abrams DB, Glasser AM, Pearson JL, et al. Harm Minimization and Tobacco Control: Reframing Societal Views of Nicotine Use to Rapidly Save Lives. Annu Rev Public Health. 2018;39:193-213.

14.	National Academies of Sciences Engineering and Medicine. Public Health Consequences of E-Cigarettes. Washington, DC; 2019.

15.	Gentzke AS, Wang TW, Cornelius M, et al. Tobacco Product Use and Associated Factors Among Middle and High School Students - National Youth Tobacco Survey, United States, 2021. MMWR Surveill Summ. 2022;71(5):1-29.

16.	Cornelius ME, Loretan CG, Wang TW, Jamal A, Homa DM. Tobacco Product Use Among Adults - United States, 2020. MMWR Morb Mortal Wkly Rep. 2022;71(11):397-405.

17.	U.S. Food and Drug Administration. FDA Notifies Companies, Including Puff Bar, to Remove Flavored Disposable E-Cigarettes and Youth-Appealing E-Liquids from Market for Not Having Required Authorization Silver Spring, MD2020 [Available from: <https://www.fda.gov/news-events/press-announcements/fda-notifies-companies-including-puff-bar-remove-flavored-disposable-e-cigarettes-and-youth>.

18.	U.S. Food and Drug Administration. FDA permits marketing of e-cigarette products, marking first authorization of its kind by the agency Silver Spring, MD2021 [Available from: <https://www.fda.gov/news-events/press-announcements/fda-permits-marketing-e-cigarette-products-marking-first-authorization-its-kind-agency>.

19.	U.S. Food and Drug Administration. FDA Issues Decisions on Additional E-Cgarette Products Silver Spring, MD2022 [Available from: <https://www.fda.gov/news-events/press-announcements/fda-issues-decisions-additional-e-cigarette-products>.

20.	U.S. Food and Drug Administration. FDA Issues Marketing Decisions on NJOY Ace E-Cigarette Products Silver Spring, MD2022 [Available from: <https://www.fda.gov/tobacco-products/ctp-newsroom/fda-issues-marketing-decisions-njoy-ace-e-cigarette-products>.

21.	Caldwell B, Burgess C, Crane J. Randomized crossover trial of the acceptability of snus, nicotine gum, and Zonnic therapy for smoking reduction in heavy smokers. Nicotine Tob Res. 2010;12(2):179-83.

22.	Thornley S, McRobbie H, Lin RB, et al. A single-blind, randomized, crossover trial of the effects of a nicotine pouch on the relief of tobacco withdrawal symptoms and user satisfaction. Nicotine Tob Res. 2009;11(6):715-21.

23.	Robichaud MO, Seidenberg AB, Byron MJ. Tobacco companies introduce 'tobacco-free' nicotine pouches. Tob Control. 2020;29(e1):e145-e6.

24.	Azzopardi D, Ebajemito J, McEwan M, et al. A randomised study to assess the nicotine pharmacokinetics of an oral nicotine pouch and two nicotine replacement therapy products. Sci Rep. 2022;12(1):6949.

25.	Lunell E, Fagerström K, Hughes J, Pendrill R. Pharmacokinetic comparison of a novel non-tobacco-based nicotine pouch (ZYN®) with conventional, tobacco-based Swedish snus and American moist snuff. Nicotine Tob Res. 2020.

26.	Stanfill S, Tran H, Tyx R, et al. Characterization of Total and Unprotonated (Free) Nicotine Content of Nicotine Pouch Products. Nicotine Tob Res. 2021;23(9):1590-6.

27.	Carpenter CM, Connolly GN, Ayo-Yusuf OA, Wayne GF. Developing smokeless tobacco products for smokers: an examination of tobacco industry documents. Tob Control. 2009;18(1):54-9.

28.	Connolly GN. The marketing of nicotine addiction by one oral snuff manufacturer. Tob Control. 1995;4(1):73-9.

29.	Rhee JU, Abugazia JY, Cruz Y, Timberlake DS. Use of internal documents to investigate a tobacco company's strategies to market snus in the United States. Tob Prev Cessat. 2021;7:11.

30.	Delnevo CD, Hrywna M, Miller Lo EJ, Wackowski OA. Examining market trends in smokeless tobacco sales in the United States: 2011-2019. Nicotine Tob Res. 2020.

31.	Marynak KL, Wang X, Borowiecki M, et al. Nicotine Pouch Unit Sales in the US, 2016-2020. JAMA. 2021;326(6):566-8.

32.	Nicokick. About Us 2020 [Available from: <https://nicokick.com/us/about-us>.

33.	Hammond H. Modern oral nicotine shows triple digit growth Chicago, IL2020 [Available from: <https://www.cspdailynews.com/tobacco/modern-oral-nicotine-shows-triple-digit-growth>.

34.	Swedish Match International. 2021 - Breaking new records, while investing in the future Stockholm, Sweden2022 [Available from: <https://www.swedishmatch.com/Our-company/Our-company-in-brief/CEO-comment/>.

35.	East KA, Reid JL, Rynard VL, Hammond D. Trends and Patterns of Tobacco and Nicotine Product Use Among Youth in Canada, England, and the United States From 2017 to 2019. J Adolesc Health. 2021;69(3):447-56.

36.	Altria Science. Underage Tobacco Use Survey 2022 [updated 2022. Available from: <https://sciences.altria.com/library/underage-tobacco-use-survey?src=topnav>.

37.	Vogel EA, Barrington-Trimis JL, Kechter A, et al. Differences in Young Adults' Perceptions of and Willingness to Use Nicotine Pouches by Tobacco Use Status. Int J Environ Res Public Health. 2022;19(5).

38.	Hrywna M, Gonsalves NJ, Delnevo CD, Wackowski OA. Nicotine pouch product awareness, interest and ever use among US adults who smoke, 2021. Tob Control. 2022.

39.	Plurphanswat N, Hughes JR, Fagerstrom K, Rodu B. Initial Information on a Novel Nicotine Product. Am J Addict. 2020;29(4):279-86.

40.	Pepper JK, Brewer NT. Electronic nicotine delivery system (electronic cigarette) awareness, use, reactions and beliefs: a systematic review. Tob Control. 2014;23(5):375-84.

41.	US Centers for Disease Control and Prevention Office on Smoking and Health. E-Cigarette Use Among Youth and Young Adults: A Report of the Surgeon General. 2016.

42.	truth Initiative. Vaporized: Youth and Young Adult Exposure to E-Cigarette Marketing. Washington, DC: Truth Initiative; 2015.

43.	Brock B, Schillo BA, Moilanen M. Tobacco industry marketing: an analysis of direct mail coupons and giveaways. Tob Control. 2015;24(5):505-8.

44.	Curry LE, Pederson LL, Stryker JE. The changing marketing of smokeless tobacco in magazine advertisements. Nicotine Tob Res. 2011;13(7):540-7.

45.	Grana RA, Ling PM. "Smoking revolution": a content analysis of electronic cigarette retail websites. Am J Prev Med. 2014;46(4):395-403.

46.	Klein EG, Berman M, Hemmerich N, et al. Online E-cigarette Marketing Claims: A Systematic Content and Legal Analysis. Tob Regul Sci. 2016;2(3):252-62.

47.	Mejia AB, Ling PM. Tobacco industry consumer research on smokeless tobacco users and product development. Am J Public Health. 2010;100(1):78-87.

48.	Sears CG, Walker KL, Hart JL, et al. Clean, cheap, convenient: promotion of Electronic cigarettes on YouTube. Tob Prev Cessat. 2017;3.

49.	Timberlake DS, Pechmann C, Tran SY, Au V. A content analysis of Camel Snus advertisements in print media. Nicotine Tob Res. 2011;13(6):431-9.

50.	Wagoner KG, Berman M, Rose SW, et al. Health claims made in vape shops: an observational study and content analysis. Tob Control. 2019;28(e2):e119-e25.

51.	Czaplicki L, Patel M, Rahman B, et al. Oral nicotine marketing claims in direct-mail advertising. Tob Control. 2021.

52.	Talbot EM, Giovenco DP, Grana R, Hrywna M, Ganz O. Cross-promotion of nicotine pouches by leading cigarette brands. Tob Control. 2021.

53.	Vassey J, Valente T, Barker J, et al. E-cigarette brands and social media influencers on Instagram: a social network analysis. Tob Control. 2022.

54.	Emery S, Kostiygina G. Exploring the amount and themes of newer smokeless tobacco product-related messages on twitter. Chicago, IL: NORC at the University of Chicago; 2022.

55.	Nicokick. The strengths of nicotine pouches 2021 [Available from: <https://nicokick.com/us/pouch-perfect/science/the-strengths-of-nicotine-pouches>.

56.	Trinkets & Trash. Zyn Point of Sale Advertisement New Brunswick, NJ2019 [Available from: <https://trinketsandtrash.org/detail.php?artifactid=14646&page=1>.

57.	Parents Against Vaping E-Cigarettes. Puffbar & Synthetic Nicotine: The Industry's Latest Tricks to Hook Our Kids. New York, NY; 2022.

58.	Patwardhan S, Fagerstrom K. The New Nicotine Pouch Category: A Tobacco Harm Reduction Tool? Nicotine Tob Res. 2022;24(4):623-5.

59.	Nicokick. What do all-white and tobacco-free mean? 2021 [Available from: <https://nicokick.com/us/pouch-perfect/what-do-all-white-and-tobacco-free-mean>.

60.	Moran MB, Heley K, Baldwin K, et al. Selling tobacco: A comprehensive analysis of the U.S. tobacco advertising landscape. Addict Behav. 2019;96:100-9.

61.	Czaplicki L, Rahman B, Simpson R, et al. Going Smokeless: Promotional Features and Reach of US Smokeless Tobacco Direct-Mail Advertising (July 2017-August 2018). Nicotine Tob Res. 2021;23(8):1349-57.

62.	Baer JS, Kivlahan DR, Donovan DM. Integrating skills training and motivational therapies. Implications for the treatment of substance dependence. J Subst Abuse Treat. 1999;17(1-2):15-23.

63.	Byron MJ, Hall MG, King JL, Ribisl KM, Brewer NT. Reducing Nicotine Without Misleading the Public: Descriptions of Cigarette Nicotine Level and Accuracy of Perceptions About Nicotine Content, Addictiveness, and Risk. Nicotine Tob Res. 2019;21(Suppl 1):S101-S7.

64.	Denlinger-Apte RL, Joel DL, Strasser AA, Donny EC. Low Nicotine Content Descriptors Reduce Perceived Health Risks and Positive Cigarette Ratings in Participants Using Very Low Nicotine Content Cigarettes. Nicotine Tob Res. 2017;19(10):1149-54.

65.	Institute of Medicine (US) Committee to Assess the Science Base for Tobacco Harm Reduction. Clearing the Smoke: Assessing the Science Base for Tobacco Harm Reduction. 2001.

66.	Johnson AC, Mays D, Villanti AC, et al. Marketing Influences on Perceptions of Reduced Nicotine Content Cigarettes. Nicotine Tob Res. 2019;21(Suppl 1):S117-s24.

67.	Mercincavage M, Saddleson ML, Gup E, et al. Reduced nicotine content cigarette advertising: How false beliefs and subjective ratings affect smoking behavior. Drug Alcohol Depend. 2017;173:99-106.

68.	National Cancer Institute. Risks associated with smoking cigarettes with low machine-measured yields of tar and nicotine. In: U.S. Department of Health and Human Services, editor. Bethesda, MD2001.

69.	Baig SA, Byron MJ, Lazard AJ, Brewer NT. "Organic," "Natural," and "Additive-Free" Cigarettes: Comparing the Effects of Advertising Claims and Disclaimers on Perceptions of Harm. Nicotine Tob Res. 2019;21(7):933-9.

70.	El-Toukhy S, Baig SA, Jeong M, et al. Impact of modified risk tobacco product claims on beliefs of US adults and adolescents. Tob Control. 2018;27(Suppl 1):s62-s9.

71.	Epperson AE, Henriksen L, Prochaska JJ. Natural American Spirit Brand Marketing Casts Health Halo Around Smoking. Am J Public Health. 2017;107(5):668-70.

72.	Gratale SK, Maloney EK, Cappella JN. Regulating language, not inference: an examination of the potential effectiveness of Natural American Spirit advertising restrictions. Tob Control. 2019;28(e1):e43-e8.

73.	Gratale SK, Maloney EK, Sangalang A, Cappella JN. Influence of Natural American Spirit advertising on current and former smokers' perceptions and intentions. Tob Control. 2018;27(5):498-504.

74.	Iles IA, Pearson JL, Lindblom E, Moran MB. "Tobacco and Water": Testing the Health Halo Effect of Natural American Spirit Cigarette Ads and Its Relationship with Perceived Absolute Harm and Use Intentions. Health Commun. 2021;36(7):804-15.

75.	O'Connor RJ, Lewis MJ, Adkison SE, Bansal-Travers M, Cummings KM. Perceptions of "Natural" and "Additive-Free" Cigarettes and Intentions to Purchase. Health Educ Behav. 2017;44(2):222-6.

76.	Andrews JC, Netemeyer RG, Burton S. Consumer generalization of nutrient content claims in advertising. Journal of Marketing. 1998;62(Oct):62-75.

77.	Sundar A, Kardes FR. The role of perceived variability and the health halo effect in nutritional inference and consumption. Psychol & Marketing. 2015;32(5):512-21.

78.	Morean ME, Bold KW, Davis DR, et al. Does it come from tobacco? Young adults' interpretations of the term "tobacco-free nicotine" in a cross-sectional national survey sample. PLoS One. 2022;17(5):e0268464.

79.	Chen-Sankey J, Ganz O, Seidenberg A, Choi K. Effect of a 'tobacco-free nicotine' claim on intentions and perceptions of Puff Bar e-cigarette use among non-tobacco-using young adults. Tob Control. 2021.

80.	Husten CG, Deyton LR. Understanding the Tobacco Control Act: efforts by the US Food and Drug Administration to make tobacco-related morbidity and mortality part of the USA's past, not its future. Lancet. 2013;381(9877):1570-80.

81.	US Food and Drug Administration. Deeming Tobacco Products To Be Subject to the Federal Food, Drug, and Cosmetic Act, as Amended by the Family Smoking Prevention and Tobacco Control Act; Restrictions on the Sale and Distribution of Tobacco Products and Required Warning Statements for Tobacco Products. Final rule. Fed Regist. 2016;81(90):28973-9106.

82.	Ashley DL, Backinger CL, van Bemmel DM, Neveleff DJ. Tobacco regulatory science: research to inform regulatory action at the Food and Drug Administration's Center for Tobacco Products. Nicotine Tob Res. 2014;16(8):1045-9.

83.	Hammond D. Tobacco packaging and labeling policies under the U.S. Tobacco Control Act: research needs and priorities. Nicotine Tob Res. 2012;14(1):62-74.

84.	World Health Organization. Guidelines for implementation of Article 11 Packaging and labelling of tobacco products Geneva, Switzerland: 2013; 2013 [Available from: <https://fctc.who.int/who-fctc/overview/treaty-instruments/packaging-and-labelling-of-tobacco-products>.

85.	Kaufman A, Klein EG, Koblitz A, Price S. A Vision for Eye-tracking Research in Tobacco Regulatory Science. Tob Regul Sci. 2018;4(6):3-7.

86.	Dillard JP, Weber KM, Vail RG. The relationship between perceived and actual effectiveness of persuasive messages: A meta-analysis with implications for formative campaign research. J Commun. 2007;57:613-31.

87.	Ferrer R, Klein WM. Risk perceptions and health behavior. Curr Opin Psychol. 2015;5:85-9.

88.	McGuire WJ. Public communication as a strategy for inducing health-promoting behavioral change. Prev Med. 1984;13(3):299-319.

89.	Noar SM, Hall MG, Francis DB, et al. Pictorial cigarette pack warnings: a meta-analysis of experimental studies. Tob Control. 2016;25(3):341-54.

90.	Portnoy DB, Ferrer RA, Bergman HE, Klein WM. Changing deliberative and affective responses to health risk: a meta-analysis. Health Psychol Rev. 2014;8(3):296-318.

91.	Sheeran P, Harris PR, Epton T. Does heightening risk appraisals change people's intentions and behavior? A meta-analysis of experimental studies. Psychol Bull. 2014;140(2):511-43.

92.	Husky R, Wilcox S, Clayton RB, Keene JR. The limited capacity model of motivated mediated message processing: meta-analytically summarizing two decades of research. Annals of the International Communication Association. 2020;44(4):322-49.

93.	Potter RF, Bolls P. Psychophysiological measurement and meaning: cognitive and emotional processing of nedia. New York: Routledge; 2012.

94.	Regmi K, Kaphle D, Timilsina S, Tuha NAA. Application of Discrete-Choice Experiment Methods in Tobacco Control: A Systematic Review. Pharmacoecon Open. 2018;2(1):5-17.

95.	Keller-Hamilton B, Fioritto M, Klein EG, et al. Visual attention to blu's parody warnings and the FDA's warning on e-cigarette advertisements. Addict Behav. 2022;125:107169.

96.	Klein EG, Quisenberry AJ, Shoben AB, et al. Health Warning Labels for Smokeless Tobacco: The Impact of Graphic Images on Attention, Recall, and Craving. Nicotine Tob Res. 2017;19(10):1172-7.

97.	Klein EG, Shoben AB, Krygowski S, et al. Does Size Impact Attention and Recall of Graphic Health Warnings? Tob Regul Sci. 2015;1(2):175-85.

98.	Leshner G, Phan L, Stevens EM, et al. Psychophysiological responses to hookah tobacco public education messages among young adults. Health Psychology. 2021;40(9):569-77.

99.	Stevens EM, Johnson AL, Leshner G, et al. People in E-Cigarette Ads Attract More Attention: An Eye-Tracking Study. Tob Regul Sci. 2020;6(2):105-17.

100.	McKee SA. Developing human laboratory models of smoking lapse behavior for medication screening. Addict Biol. 2009;14(1):99-107.

101.	McKee SA, Krishnan-Sarin S, Shi J, Mase T, O'Malley SS. Modeling the effect of alcohol on smoking lapse behavior. Psychopharmacology (Berl). 2006;189(2):201-10.

102.	Stevens EM, Johnson A, Wander N, et al. Influence of regulated vs. unregulated e-cigarette advertising on e-cigarette use: A laboratory vaping lapse task study. International Communication Association 2020 Annual Conference; Virtual2020.

103.	Maiese DR, Hendershot TP, Strader LC, et al. PhenX—Establishing a Consensus Process to Select Common Measures for Collaborative Research. 2013.

104.	Piper ME, Brown DC, Hendershot TP, Swan GE, Group PTHSCW. PhenX: Host: Social/Cognitive measures for tobacco regulatory research. Tob Control. 2020;29(Suppl 1):s5-s12.

105.	Unger JB, Chaloupka FJ, Vallone D, et al. PhenX: Environment measures for Tobacco Regulatory Research. Tob Control. 2020;29(Suppl 1):s35-s42.

106.	Williams VF, Smith AA, Villanti AC, et al. Validity of a Subjective Financial Situation Measure to Assess Socioeconomic Status in US Young Adults. J Public Health Manag Pract. 2017;23(5):487-95.

107.	Klein EG, Quisenberry AJ, Shoben AB, Romer D, Peters E. The Influence of Health Numeracy and Health Warning Label Type on Smoking Myths and Quit-Related Reactions. Nicotine Tob Res. 2019;21(7):974-8.

108.	Haun J, Luther S, Dodd V, Donaldson P. Measurement Variation Across Health Literacy Assessments: Implications for Assessment Selection in Research and Practice. Journal of Health Communication. 2012;17(sup3):141-59.

109.	Lochbuehler K, Mercincavage M, Tang KZ, et al. Effect of message congruency on attention and recall in pictorial health warning labels. Tob Control. 2017.

110.	Lochbuehler K, Tang KZ, Souprountchouk V, et al. Using eye-tracking to examine how embedding risk corrective statements improves cigarette risk beliefs: Implications for tobacco regulatory policy. Drug Alcohol Depend. 2016;164:97-105.

111.	Strasser AA, Tang KZ, Romer D, Jepson C, Cappella JN. Graphic warning labels in cigarette advertisements: recall and viewing patterns. Am J Prev Med. 2012;43(1):41-7.

112.	Clayton RB, Leshner G, Bolls PD, Thorson E. Discard the Smoking Cues-Keep the Disgust: An Investigation of Tobacco Smokers' Motivated Processing of Anti-tobacco Commercials. Health Commun. 2017;32(11):1319-30.

113.	Leshner G, Bolls P, Thomas E. Scare' em or disgust 'em: the effects of graphic health promotion messages. Health Commun. 2009;24(5):447-58.

114.	Leshner G, Cheng IH. The effects of frame, appeal, and outcome extremity of antismoking messages on cognitive processing. Health Commun. 2009;24(3):219-27.

115.	Mays D, Villanti A, Niaura RS, Lindblom EN, Strasser AA. The Effects of Varying Electronic Cigarette Warning Label Design Features On Attention, Recall, and Product Perceptions Among Young Adults. Health Commun. 2019;34(3):317-24.

116.	Kaufman AR, Twesten JE, Suls J, et al. Measuring Cigarette Smoking Risk Perceptions. Nicotine Tob Res. 2020;22(11):1937-45.

117.	Lipkus IM, Mays D. Comparing harm beliefs and risk perceptions among young adult waterpipe tobacco smokers and nonsmokers: Implications for cessation and prevention. Addict Behav Rep. 2018;7:103-10.

118.	Lipkus IM, Mays D, K PT. Characterizing Young Adults' Susceptibility to Waterpipe Tobacco Use and Their Reactions to Messages About Product Harms and Addictiveness. Nicotine Tob Res. 2017;19(10):1216-23.

119.	Mays D, Johnson AC, Phan L, et al. Tailored mobile messaging intervention for watepripe tobacco cessation in young adults: a randomized trial. Am J Public Health. 2021;Online Ahead of Print:e1-e10.

120.	Mays D, Johnson AC, Phan L, et al. Effect of risk messages on risk appraisals, attitudes, ambivalence, and willingness to smoke hookah in young adults. Health Psychol Behav Med. 2020;8(1):96-109.

121.	Mays D, Johnson AC, Glasser A, Mercincavage M, Strasser AA. Effects of IQOS health warnings and modified risk claims among young adult cigarette smokers and non-smokers. Tob Control. 2021.

122.	Mays D, Moran MB, Levy DT, Niaura RS. The Impact of Health Warning Labels for Swedish Snus Advertisements on Young Adults' Snus Perceptions and Behavioral Intentions. Nicotine Tob Res. 2016;18(5):1371-5.

123.	Phan L, Strasser AA, Johnson AC, et al. Young Adult Correlates of IQOS Curiosity, Interest, and Likelihood of Use. Tobacco Regulatory Science. 2020;6(2):81-90.

124.	Salloum RG, Haider MR, Barnett TE, et al. Waterpipe Tobacco Smoking and Susceptibility to Cigarette Smoking Among Young Adults in the United States, 2012-2013. Prev Chronic Dis. 2016;13:E24.

125.	Roberts ME, Ferketich AK. Hookah Susceptibility and Transitions Over the First Year of College. J Stud Alcohol Drugs. 2020;81(2):195-202.

126.	Loukas A, Marti CN, Cooper M, Pasch KE, Perry CL. Exclusive e-cigarette use predicts cigarette initiation among college students. Addict Behav. 2018;76:343-7.

127.	Barrington-Trimis JL, Liu F, Unger JB, et al. Evaluating the predictive value of measures of susceptibility to tobacco and alternative tobacco products. Addict Behav. 2019;96:50-5.

128.	Perez A, Bluestein MA, Kuk AE, et al. Age of Onset of Susceptibility to Different Tobacco Products Among Non-Susceptible US Young Adults: Findings from the Population Assessment of Tobacco and Health Study Waves 2-4 (2014-2017). Tob Use Insights. 2021;14:1179173X211065643.

129.	Kasza KA, Ambrose BK, Conway KP, et al. Tobacco-Product Use by Adults and Youths in the United States in 2013 and 2014. N Engl J Med. 2017;376(4):342-53.

130.	Kasza KA, Borek N, Conway KP, et al. Transitions in Tobacco Product Use by U.S. Adults between 2013⁻2014 and 2014⁻2015: Findings from the PATH Study Wave 1 and Wave 2. Int J Environ Res Public Health. 2018;15(11).

131.	Ling PM, Glantz SA. Using tobacco-industry marketing research to design more effective tobacco-control campaigns. JAMA. 2002;287(22):2983-9.

132.	Ling PM, Glantz SA. Why and how the tobacco industry sells cigarettes to young adults: evidence from industry documents. Am J Public Health. 2002;92(6):908-16.

133.	Villanti AC, Niaura RS, Abrams DB, Mermelstein R. Preventing Smoking Progression in Young Adults: the Concept of Prevescalation. Prev Sci. 2019;20(3):377-84.

134.	Green AE, Mays D, Falk EB, et al. Young Adult Smokers' Neural Response to Graphic Cigarette Warning Labels. Addict Behav Rep. 2016;3:28-32.

135.	Klein EG, Quisenberry AJ, Shoben AB, et al. Testing a Brief Web-based Intervention to Increase Recognition of Tobacco Constituents. Tob Regul Sci. 2018;4(6):83-94.

136.	Stevens EM, Villanti AC, Leshner G, et al. Integrating Self-Report and Psychophysiological Measures in Waterpipe Tobacco Message Testing: A Novel Application of Multi-Attribute Decision Modeling. Int J Environ Res Public Health. 2021;18(22).

137.	Klein EG, Czaplicki L, Berman M, Emery S, Schillo B. Visual Attention to the Use of #ad versus #sponsored on e-Cigarette Influencer Posts on Social Media: A Randomized Experiment. J Health Commun. 2020;25(12):925-30.

138.	Klein EG, Alalwan MA, Pennell ML, et al. Waterpipe Warning Placement and Risk Perceptions: An Eye Tracking Study. Am J Health Behav. 2021;45(1):186-94.

139.	Strasser AA, Cappella JN, Jepson C, et al. Experimental evaluation of antitobacco PSAs: effects of message content and format on physiological and behavioral outcomes. Nicotine Tob Res. 2009;11(3):293-302.

140.	Phan L, Villanti AC, Leshner G, et al. Development and Pretesting of Hookah Tobacco Public Education Messages for Young Adults. Int J Environ Res Public Health. 2020;17(23).

141.	Keller-Hamilton B, Stevens EM, Villanti A, et al. Messaging to prevent and reduce young adults' waterpipe tobacco smoking: A randomized trial. Addict Behav. Under Review.
